# A Robust Self-Powered Triboelectric Sensor for Risk Mitigation in Seismic Scenarios: IoT Communication and Dimensional Monitoring

**DOI:** 10.1021/acsomega.6c00458

**Published:** 2026-06-11

**Authors:** José Sánchez del Río Sáez, Antonio Vázquez-López, Jorge Edison Pozo Benavides, Alba López Laguna, Martin Andolfi, Rafael Cascón, Francisco Santos Olalla, Sofía Paramio, Yolanda Ballesteros, Carlos Cruz, Vanesa Martínez, José Luis Jiménez, José Benito Bravo Monge, Xiang Ao, De-Yi Wang

**Affiliations:** † 16771 Universidad Politécnica de Madrid (UPM), E.T.S. de Ingeniería y Diseño Industrial, C/Ronda de Valencia 3, 28012 Madrid, Spain; ‡ IMDEA Materials Institute, C/Eric Kandel, 2, Getafe, Madrid 28906, Spain; § Universidad Pontificia de Comillas, Mechanical Engineering Department, Alberto Aguilera 25, 28015 Madrid, Spain; ∥ Materials Science and Engineering Area, Escuela Superior de Ciencias Experimentales y Tecnología, 16776 University Rey Juan Carlos, Tulipán Street, Móstoles 28933, Madrid, Spain; ⊥ Institute for Research in Technology, Mechanical Engineering Department, Universidad Pontificia de Comillas, Alberto Aguilera 25, 28015 Madrid, Spain; # 16720 University of Alcala, Department of Electronics, Alcala de Henares, 28871 Madrid, Spain; ¶ Space Applications, Geophysics and Astronomy Division. General Subdirection, 73052 Instituto Geográfico Nacional (IGN). C/General Ibáñez de Ibero, 3. 28003 Madrid, Spain

## Abstract

Currently, there is an increasing need for low-cost detectors that can measure ground motion with high sensitivity and selectivity. Triboelectric nanogenerators (TENGs) have arisen as low-cost self-powering sensing devices that can be used in multiple applications that involve vibration and motion, such as in earthquake detection. In this work, a TENG-based seismic device (SEISTENG) is designed with the purpose of detecting either 2D or 3D vibrating motion. This device is based on low-cost TENGs and comprises the walls of a 3D-printed polylactic acid box with a sliding metal ball inside and rolling on its horizontal base. The TENG transducer dynamical properties for a high-frequency range (0.5–50 Hz), long duration operation, and robustness were measured. The SEISTENG was validated by simulating the 1995 Kobe earthquake on a biaxial vibration table and the 2011 Lorca earthquake on a triaxial system, demonstrating its ability to detect seismic excitation signals with high accuracy (2D or 3D SEISTENG). The technology produced a response comparable to that of the commercial piezoelectric sensor D220-A4BR-1305YB, and its signals could be monitored remotely in real time using an FPGA-based STEMlab board, a LabVIEW interface, and Internet of things (IoT) platforms.

## Graphical abstract

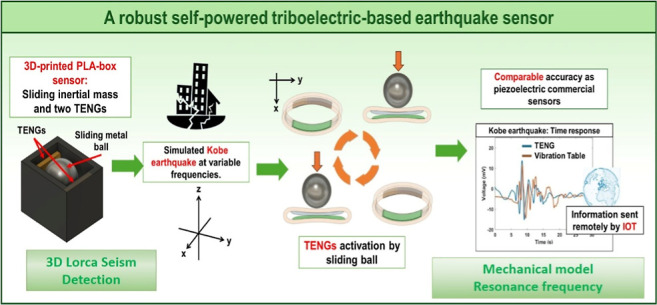

## 1 Introduction

The development of new technologies with the capability of detecting oscillating signals constitutes one of the main challenges nowadays.^1,2^ Researchers in electronics and communication engineering are seeking with urgency the design and commercialization of such devices^3–5^ ideally aiming for sustainable materials and low costs.^6,7^

Low-cost novel and integrated sensors connected to high-resolution data acquisition systems (DAQs) will have a significant impact on several scientific fields: edification,^8,9^ natural catastrophe monitoring,^10,11^ biology,^12,13^ or medicine.^14,15^ Their arrival will reduce the high costs related to the fabrication of expensive broad networks with their sensors located either locally or globally in wider areas.

A specific set of sensors, which could be beneficiated from the development of cheap and reliable wide-broadband technologies, are among^16–18^ the seismic sensors. Most of them measure magnitudes such as displacement, velocity, and acceleration, which are essential variables in monitoring, for instance, ground vibrations.^19,20^ Furthermore, these sensors can be used to mitigate possible risks associated with abrupt ground motion episodes and will allow preventing huge disaster population that might suffer. As a result, these sensors fall into the category of seismic design.^21^

The triboelectric nanogenerators (TENGs) are a self-powered, sustainable, and low-cost technology that have been announced among the top ten emerging technologies in chemistry in 2024.^22^ Triboelectric nanogenerators (TENGs) have experienced remarkable and continuous development since their discovery in 2012 by Prof. Zhong Lin Wang and colleagues, leading to a rapidly expanding and highly regarded research field.^23,24^ The main principle of TENGs is widely known and is described elsewhere,^25–27^ which, in summary, is based on the opposite electronegative layers fabricated with different materials that enhance charge transfer between them and produce self-electric energy when they are in dynamic friction or approaching one another.

In our previous paper, we were able to design an accurate and wide broadband seismic sensor, but it was only deployed on the *Z* axis by placing a load over the TENG.^4^

Recent advances^28–30^ have facilitated the development of cost-effective three-dimensional seismic monitoring solutions based on triboelectric nanogenerators (TENGs).^31^ Addressing the demand for low-cost, broadband, and multidirectional seismic sensing, TENG technology is proposed here as a viable platform for motion detection in the X–*Y* plane and along the *Z* axis, the latter previously validated by the authors.^4,11^ TENGs have demonstrated their suitability for decentralized safety systems and ambient-motion risk mitigation, as well as high-performance vibration mode control in practical environment.^32–35^ Additionally, long-term robustness considerations can be informed by comprehensive studies on degradation mechanisms and durable triboelectric materials,^36,37^ reinforcing the potential of SEISTENG architectures for real-world deployment.

A further advantage of TENG systems lies in their compatibility with Internet-of-Things (IoT) infrastructures,^38–42^ enabling in situ and real-time remote monitoring. Such capabilities have already been demonstrated in related domains, including biomedical sensing,^43,44^ while recent work has confirmed that LoRa communication protocols can support ground-motion monitoring.^11^ Depending on the system architecture, TENG signals may be processed locally or transmitted to cloud platforms for enhanced analysis.

The applicability of TENGs to sensing natural phenomena, particularly seismic oscillations, has been demonstrated in several recent studies.^45–52^ A friction-free levitating oscillator TENG reported in^45^ produced measurable electrical outputs under simulated vibration conditions, although its response to slow or continuous frequency signals was limited. A lotus-inspired early warning TENG presented in^46^ effectively converted small seismic excitations into electrical energy while offering environmental robustness, yet broadband frequency detection remained constrained. The “Smart Maracas” device in^47^ exhibited strong linear correlation between the output voltage and acceleration across three axes but was unable to resolve slow or mixed-frequency inputs. Similarly, a hybrid triboelectric–electromagnetic generator described in^48^ enabled self-powered seismic detection over multiple frequency ranges, though low-frequency sensitivity was reduced.

Additional efforts have been focused on structural and displacement monitoring applications. A hydrogel-based TENG sensor in ref 49 successfully captured plastic hinge deformation in structural elements without directly measuring force or acceleration. Displacement-oriented TENG systems proposed for damper monitoring^50^ and landslide detection^51^ demonstrated good linearity yet lacked full multidimensional sensing capability and sufficient electrical output. A spherical inertial TENG sensor introduced in ref 52 enabled the measurement of angular and linear motion but similarly showed limitations in detecting low-frequency or continuous signals. In addition, TENG sensitivity enhancement is needed in cases where vibrations are low and this was presented by Youngwook Chung et al. in ref 53 where the incorporation of the Ti packaging leads to a significant increase in triboelectric power density, up to 310% compared with the absence of it when measured under a tissue-mimicking material, and this enables long-term stability and Bluetooth communication in vivo. Furthermore, a demonstration of the charge effect in water is presented in ref,^54^ where efficient in situ disinfection in a portable water bottle by directly harvesting the electrostatic charges induced by walking to stimulate electroporation is presented.

Collectively, these studies highlight the expanding role of the TENG technology in seismic and structural monitoring while underscoring persistent challenges in broadband and multidirectional sensing, thereby motivating the present work. It is important to note that an induction effect may arise when a charged mass approaches a TENG device without direct contact between triboelectric layers, a phenomenon that must be considered in sensor interpretation. The recent progress of TENG-based sensing platforms for monitoring dynamic phenomena has partially motivated the present work.^5,55–57^

In contrast to the previously reported one-dimensional configuration (1D-SEISTENG), the design proposed here employs a 3D-printed enclosure and a free-standing inertial mass that slides within the structure. After appropriate calibration, this configuration enables symmetric monitoring of motion in the X–*Y* plane, thereby extending the current state of the SEISTENG technology. The resulting device, termed 2D-SEISTENG, exploits triboelectric interactions produced by inertial impacts against the base of a polylactic acid (PLA) structure, enabling accurate reconstruction of seismic excitation spectra in both time and frequency domains under simulated conditions. Validation was performed using a bidirectional vibration platform reproducing the characteristics of the 1995 Kobe earthquake.

Building upon this architecture, a three-dimensional configuration (3D-SEISTENG) is proposed to enable sensing along the X, Y, and *Z* axes. Its modular structure incorporates horizontal sensing units aligned with orthogonal directions in the *X*–*Y* plane and a vertical sensing unit, allowing multidirectional seismic monitoring. The system was evaluated on a triaxial vibration platform simulating the dynamic characteristics of the 2011 Lorca earthquake, demonstrating its capability for a realistic three-axis seismic detection.

## 2 Materials and Methods

### 2.1 Fabrication of the TENG Materials

The TENG employed in this work consisted of two different layers: one of poly­(vinyl alcohol) (PVA) and the other one composed of polydimethylsiloxane (PDMS). In addition, Cu paper was included to fix the electrodes. Details regarding fabrication and materials employed in this type of TENG can be found elsewhere.^58^ Variable thicknesses of the PDMS layer were fabricated (1, 2, and 4 mm). The whole size of each TENG is 40 mm × 37 mm, while the active area (the size of the PVA layer and PDMS films) is 30 mm × 35 mm. The PDMS layer thickness used for sensing is of 1 mm. A 2-layer piezoelectric actuator and sensor D220-A4BR-1305YB (Figure S1) from^59^ was also used to compare the TENGs integrated in the seismic prototype. Difference of sensitivity due to thickness was studied previously by the authors in.^5^

### 2.2 Vibration Tables for Simulating Realistic Seismic Events

A vibration table from the Matest model C278 (Figure 1a) (from Industrial Engineering Technical Superior School—ETSII-) with dimensions of 600 × 400 mm and a maximum frequency of 3000 rpm was used to generate vibrations in the frequency range of 1–5 Hz under different displacements of the prism box (Figure 1b) with the metal sphere inside. The mass (546.7 ± 0.8 g) and radius (50.7 ± 0.1 mm) of this metal sphere were measured, and this is shown in Tables S1–S3, the same as the displacement length of the metal sphere and the TENG stiffness (151 ± 10 N/m). Furthermore, a V20 shaker (Figure 1c) powered with a PA100 amplifier and connected to a vibration table (Figure 1d) was used to generate up to 8.3 Hz of frequency pulses with different excitation voltage amplitudes (up to 20 V).

**Figure 1:**
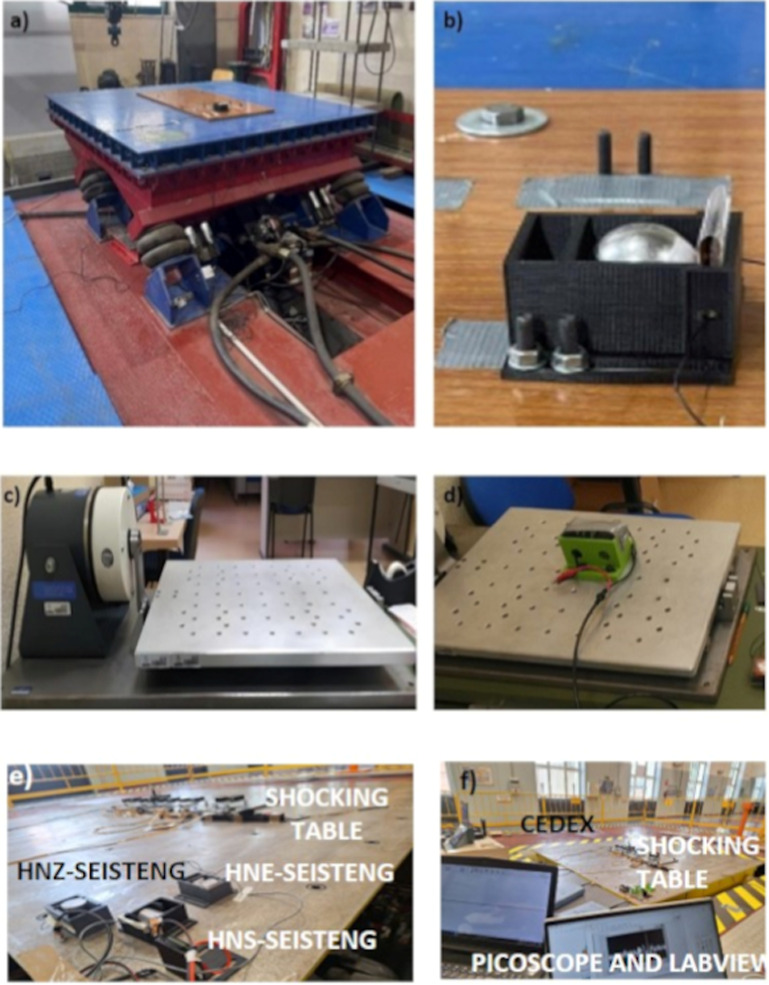
(a) A vibration table from Matest, model C278, on which (b) the TENG case with the TENG inside and the inertial mass is shown; (c) V20 shaker powered with a PA100 amplifier on which (d) the TENG is screwed; (e) different parts of 3D-SEISTENG fixed to the shocking table; and (f) software and setup employed in monitoring the different seismic waves.

In addition, a 3-axis shaking table owned by CEDEX (Centro de Estudios y Experimentación de Obras Públicas) was used to monitor oscillations artificially generated (Figure 1e,f). A lower earthquake that took place in Lorca, Murcia, Spain, was simulated. 2D-SEISTENG and 3D-SEISTENG different directional components were screwed to a metal board placed on the shaking table, and the simulated earthquake could be detected by TENG transducers in the three directions North-East (NE), North-south (NS), and North-Z (NZ) directions. A magnified image of the components fixed in the triaxial vibrating table and the setup is shown in Figure S2.

### 2.3 Data Acquisition Systems

A Picoscope 2000 oscilloscope with Picoscope 7 software was programmed to measure at 200 MSamples/s in intervals of 10 s, with the software used to filter the signal up to 1 Hz (low-pass filtering) in order to eliminate high-frequency noise. This DAQ was also used to detect induction phenomena produced in the TENGs when the inertial mass moved toward or away from them and when measuring different vibration frequencies.

A conventional ISO-TECH (IDS 6052-U) digital oscilloscope from the ETSIDI (Escuela Técnica Superior de Ingeniería y Diseño Industrial) and DP2012B Tektronix one from IMDEA Materials were also used to measure induced and direct TENG signals.

### 2.4 Software and Programs Used

To analyze results obtained from the TENG placed in the seismic prototype described above that received impacts from the inertial mass due to the motion of the Matest shock table when simulating the Kobe earthquake, the signal analysis tools from MATLAB Toolbox were used to filter and smooth the TENG voltage signals. The fast Fourier transform (FFT) function was employed in a software programmed in MATLAB by the authors, and the first harmonic was chosen again for signal analysis. Spectrograms and scalograms were also plotted with the same MATLAB analysis tool in order to compare the TENG signals in time and frequency domains to those programmed for simulating the Kobe earthquake in the ETSII vibration table. Furthermore, raw data were used when calculating the FFT, which should only affect the harmonics amplitudes if data are filtered but not to their frequency values. In addition, with the purpose of simulating the effect of compression forces in the TENG layers when they are in contact and with a small preload, the basic equation for the contact-mode TENG is used and solved as reported in ref 5. For this, Google Colab and Python language were used for software compilation and execution.

### 2.5 2D-SEISTENG Sensor Prototype Manufacturing

In order to measure the induction effect when the inertial mass is approaching the TENG and causing after traction and compression, a PLA prism box with dimensions of 20 cm long, 10 cm wide, and 10 cm high was fabricated with a one head MK3S+ Prusa 3D printer. Either a polished metal cylinder of 9 cm of diameter and 15 cm long or a metal sphere of 7 cm of diameter, both with the same weight, was placed inside the box with the aim of sliding on the base under motion (see Figure 2a,b). This motion was produced by a shake table that vibrated at different frequencies generated by a V20 shaker powered with a PA100 amplifier at the aforementioned ETISIDI (Figure 2c). This table was used to generate up to 20 Hz of frequency pulses with different voltage amplitudes (up to 20 V). Furthermore, the Quanser Shake table (see Figure 2d), owned by ICAI (Comillas Pontifical University: Superior Technical School of Engineering), was used to study the TENG response for different external frequencies, elongations, and forces by an interface programmed with MATLAB. In this last case, two TENGs were glued on the opposite parallel walls, most separated, and that marked the direction of the motion. When measuring slower motion without any impact, several TENGs were stacked together at both sides of the box so that they began the motion with an initial preload (Figure 2b), although only one of them at each side was used as a transducer.

**Figure 2:**
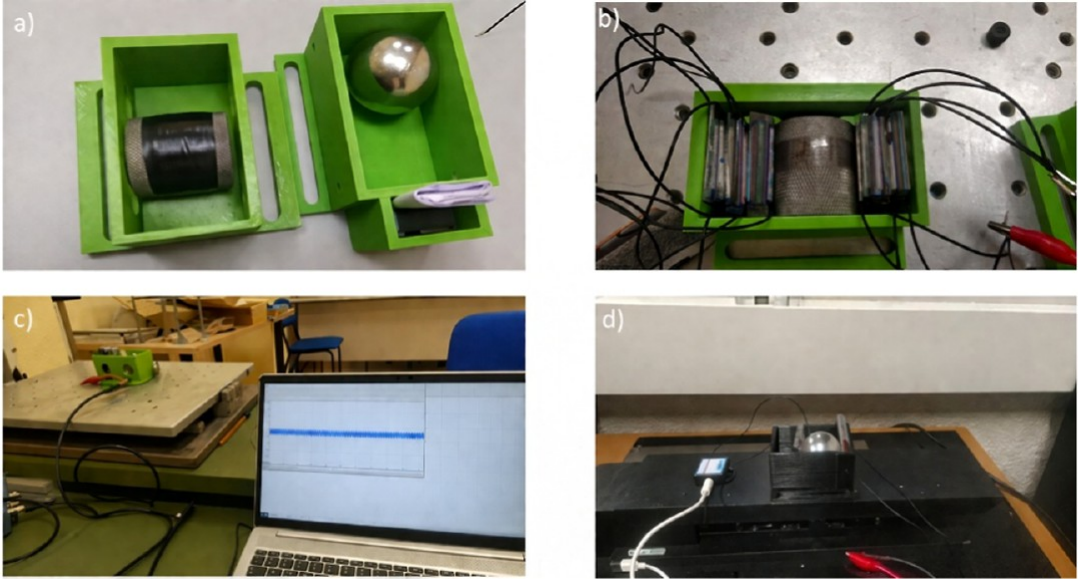
(a) Inertial masses (metal sphere and cylinder) used in the 3D printed box; (b) TENGs placed in the walls of the box with the cylinder in the middle exerting an initial preload on them; (c) vibration table and Picoscope DAQ and software; and (d) Quanser vibration table with the metal sphere inside the 3D printed case.

According to the X/Y prototype, a novel one was designed and fabricated in order to measure vibrations in three directions of the space. As can be seen later, the prototype has two in-plane prism boxes with an inertial mass rolling along each of the motion directions (X or Y) and two cubical boxes in one of the corners of the box, on where a TENG is inside of it and placed on its base with a weight of 0.5 kg resting on its triboelectric layers. This last receptacle is designed in such a way that Z-vibration motion can be monitored, and the prototype conforming to this is named 3D-SEISTENG.

### 2.6 Calibration of the TENGs Used in 2D-SEISTENG

A two-layer piezoelectric actuator and sensor D220-A4BR-1305YB from PIEZO.COM were used to calibrate the TENGs of 2D-SEISTENG. For this, TENG and piezoelectric sensors were crabbed between the jaws of an INSTRON mechanical traction/compression machine, which was programmed to move at different velocities (Figure S1a,b). First, only the piezoelectric sensor was calibrated for velocity and acceleration up to forces of 1 N and a jaw elongation of 0.2 mm. Then, for the TENG calibration, the TENG together with the piezoelectric sensor were inserted too between the two jaws but one separated from the other so that force and elongation could be monitored by the INSTRON software while the user was compressing/releasing the TENG-Piezo system with the hand.

### 2.7 Electrical Characterization of 2D-Seisteng

Electrical voltage with a Picoscope oscilloscope and electrical current with a low current LCM600T amplifier were measured in order to evaluate the open circuit voltage Voc and the shortcut current Isc, respectively, for different frequencies (1–5 Hz) of the vibrating table on which the 2D-SEISTENG is fixed. In addition, voltage and current generated by the TENG for the same frequencies were measured too for different loads (1 Ω – 1 GΩ). These results allowed calculating the maximum electrical power generated for this frequency range.

### 2.8 The Force Damping Excitation Model Used to Explain the TENG Seismic Sensor Excitation

Although at first sight, it seems that it is the seismic sensor that operates as a classical vibro-impact oscillator, it actually operates as a force-damped oscillator. This is explained by J. Sánchez del Río et al. in Section 3 of the Supporting Information of ^5^. In this work, an improved model is presented with the real values of the elastic constant, the damping constant, and the inertial mass (see Section S3). SEISTENG model has a mass m, spring k, and viscous damper c connected to a structure that moves on the ground. The mechanical quantity of interest is usually the relative displacement of the mass with respect to the base because it drives the transducer.

Motion equation is described as

1 mẍ+c(ẋ−ẏ)+k(x−y)=0

According to the definition of displacement z, the equation can be written as

2 m×z̈+c×ż+k×z=−mÿ

If the Laplace transform is applied and considering position initial conditions and zero velocity

3 $$\left(ms^{2}+cs+k\right)Z\left(s\right)=-ms^{2}Y\left(s\right)$$

If base acceleration is defined as Abase­(t) = ÿ(t), and considering null initial conditions

$$Abase\left(s\right)=s^{2}Y\left(s\right)$$

A direct expression between Z(s) and Abase (s) is obtained

4 $$\frac{Z\left(s\right)}{Abase\left(s\right)}=-\frac{m}{\left(ms^{2}+cs+k\right)}$$

The frequency response is obtained by evaluating the transfer function on the imaginary axis, that is, by substituting s = jω. It is defined as

5 $$H\left(j\omega \right)=\frac{Z\left(j\omega \right)}{Abase\left(j\omega \right)}=-\frac{m}{\left(k-m\omega^{2}+jc\omega \right)}$$

The mathematical model is developed thoroughly in Section 3. The magnitude and phase are defined as

6 $$\left|H\left(j2f\pi \right)\right|=m\frac{1}{\sqrt{{\left(k-m{\left(2f\pi \right)}^{2}\right)}^{2}+{\left(c2f\pi \right)}^{2}}}$$

7 $$∠H\left(j2f\pi \right)=180^{{}^{\circ}}-\arctan\left(\frac{c2f\pi }{k-m{\left(2f\pi \right)}^{2}}\right)$$

In this work, a fraction of the critical damping ζ = 0.005 was used, typical of steel springs.

Applying the magnitude and phase equations, with the conditions of K = 150.9 N/m and a mass m = 0.5467 kg. The viscous damping coefficient is obtained using the following expression

8 $$c=2\zeta \sqrt{2\cdot km}$$

$$c=20.005\sqrt{150,9\cdot 0,5}=0,00868N\cdot s/m$$

By substituting values into the magnitude and phase equations, the following formulations are obtained

9 $$\left|H\left(j2f\pi \right)\right|=0.5\frac{1}{\sqrt{{\left(150,9-0,5467{\left(2f\pi \right)}^{2}\right)}^{2}+{\left(2\cdot 0,00868f\pi \right)}^{2}}}$$

10 $$∠H\left(j2f\pi \right)=180^{{}^{\circ}}-\arctan\left(\frac{2\cdot 0,00868f\pi }{150,9-0,5467{\left(2f\pi \right)}^{2}}\right)$$

The solutions are plotted with MATLAB for the 2D-SEISTENG detecting the KOBE simulated earthquake (Figure S18).

### 2.9 Analysis Software for Induction Detection and FFT Calculation

A MATLAB program designed by the authors was used to plot a voltage (V) generated signal and allowed the determination of slow oscillations due to electric induction produced by the metal sphere rolling inside the box. A similar filter to that used in the Picoscope to compare the frequency spectrum (low pass filter) was used. This is known as the moving average function that is dependent on the parameter α, which is less than 1. Often, α = 0.01 was chosen and represents the cutoff frequency used in a standard low pass frequency filter.

In addition, to analyze results obtained from the TENG placed in the seismic prototype described above and receiving impacts from the inertial mass due to the motion of the Matest shock table when simulating the Kobe earthquake, signal analysis tools from MATLAB Toolbox were used to filter and smooth the TENG voltage signals. The FFT function was employed in a software programmed in MATLAB by the authors, and the first harmonic was chosen again for signal analysis. Spectrograms and scalograms were plotted with the same MATLAB analysis tool in order to compare the TENG signals in the time and frequency domains to those programmed for simulating the Kobe earthquake in the ETSII vibration table.

Furthermore, DC suppression, use of the Hanning window, calculation of the FFT, FFT logarithmic scale plots, frequency peak identification, logarithmic scale, power spectral density (PSD), signal filtering, calculation of the PSD, horizontal-to-vertical spectral ratio (HSVR), HSVR smoothing with the Konno–Ohmach method, and dominant frequency of the area detected were performed with Python scripts run in the Anaconda online platform to analyze seism spectra obtained with 3D-SEISTENG. In order to calculate the horizontal-to-vertical spectral ratio (HSVR), three files corresponding to each of the three axes used in the seism detection were used. These components are named with the capital letters: North-East hemisphere (HNE), North-South hemisphere (HSE), and North Z hemisphere (HZE) and correspond to the NE, SE, and vertical to the plane (shaking table) directions where 3D-SEISTENG transducers are positioned along them. The HSVR is defined as follows

11 $$HSVR\left(f\right)=\frac{H\left(f\right)}{\left|HNZ\left(f\right)\right|}$$

where H­(*f*) is the amplitude spectrum of each component.

12 $$H\left(f\right)=\sqrt{\frac{{\left|HNE\left(f\right)\right|}^{2}+{\left|HNN\left(f\right)\right|}^{2}}{2}}$$

The horizontal-to-vertical spectral ratio (HVSR), or horizontal-to-vertical spectral ratio, is a passive geophysical method (called the Nakamura method) that analyzes ambient seismic noise to determine the fundamental resonance frequency of the ground. As can be seen from previous equations, it is calculated by dividing the Fourier spectrum of the horizontal components of the ground motion by the spectrum of the vertical component, and the resulting peak indicates the natural frequency of the site.

In addition, the PSD will be used to determine the energy density of the seism. It shows how the energy of a signal is distributed as a function of frequency and it is defined as follows

13 $$PSD=\frac{Power}{Hz}$$

Furthermore, power is defined as follows: when FFT is applied, we can define spectral power for each frequency as

14 $$Power={\left|X\left[k\right]\right|}^{2}$$

where *X*[*k*] is the FFT of x­[n], with x­[n] being a discrete signal with N samples.

In addition, if the fundamental resonance frequency is f_0_, an estimate of sediment thickness H is

15 $$f_{0}=\frac{V_{s}}{H}$$

where Vs is the average shear-wave velocity (m/s) and H is the thickness of soft sediments.

### 2.10 Transmission of the Voltage TENG Signal to the Cloud

A STEMLab 125–14 EDU PACK (Red Pitaya) with a sensing array for multiple detection capabilities and an information processing built around an ARM processor and a field programmable gate array is used to transmit remote voltage TENG signals to the cloud. The DAQ is based on an SoC that already integrates the ARM cortex along with the Zynq 7000 FPGA (see Figure 6a). The readout is performed by incorporating a 14 bits ADC able to convert and acquire up to 125MS/s. A buffer is also implemented with a pointer indicating the position being read in each iteration. It also incorporates different peripherals for digital communication as they are Ethernet, USB, I2C, SPI, UART, and some general input–output GPIO pins. The LabVIEW visual programming environment was used to monitor signals sent via Wi-Fi and received. TENG electrodes were coupled directly to the board, and a visual programming environment, which is deployed by LabVIEW software, was implemented to visualize it in the network. Standard commands for programmable instrumentation (SCPI) are used to enable acquisition when the SCPI server is started and the IP address is automatically assigned.

Amplitude accuracy was evaluated using calibrated gain and offset values. In particular, the board was configured in low-voltage (LV) mode, which offers improved measurement precision and higher resolution by minimizing signal attenuation and allowing direct input ADC. Within the frequency range of 0–30 Hz, the standard deviation measured for a 1 V DC input was 0.002 V. The trigger system also plays a critical role in ensuring accurate data acquisition. It was configured to initiate sampling when the input signal crosses a predefined voltage threshold in the rising direction. In this setup, the trigger was initiated manually via the LabVIEW interface. Furthermore, to address network latency and synchronization issues (the board operates remotely via WiFi, while LabVIEW runs locally), a buffering mechanism was implemented (6.384 samples) to ensure reliable data transfer without sample loss and a 500 ms TCP timeout was set to handle communication delays effectively.

## 3 Results and Discussion

### 3.1 Working Principle of the 2D-Seisteng

During ground motion, positive or negative pulses are generated during the tensile/compression process, and they are dependent on the TENG layer thickness, relative velocity of the tribolayers, and separating distance. A sketch of the seismic sensor working principle proposed as with the prototype described in ref 5 with the inertial mass giving impacts with the walls of a prism box is shown in Figure 3c,e. Here, two TENGs generate electrical power alternatingly (TENG 1 and TENG 2) because they are positioned one in front of the other separated by the length of the box. After an initial state (Figure 3b,b′ and d,d′) when the ground moves to the right (or to the left, respectively), ideally, the inertial mass tends to be in the same position by compressing the left TENG while the right one is released (Figure 3c,c′). In this situation, a voltage pulse is generated by the left TENG, while the right TENG is in a static mode and charges of the triboelectric layers are not moving. Now, the seismic sensor again remains static (or the inertial mass does not touch any of the tribolayers); there is neither motion of the ground (or no force is exerted on the TENGs) nor any voltage signal generated (Figure 3d)). Then, the ground moves to the left (or the right TENG receives the impact of the inertial mass), and as a result, the opposite dynamic mode is activated: the right TENG is compressed while the left one has already been released (Figure 3e)).With this configuration, the frequency of the TENG signals generated is twice the excitation frequency when both the left and the right TENGs are considered as a whole system.

**Figure 3:**
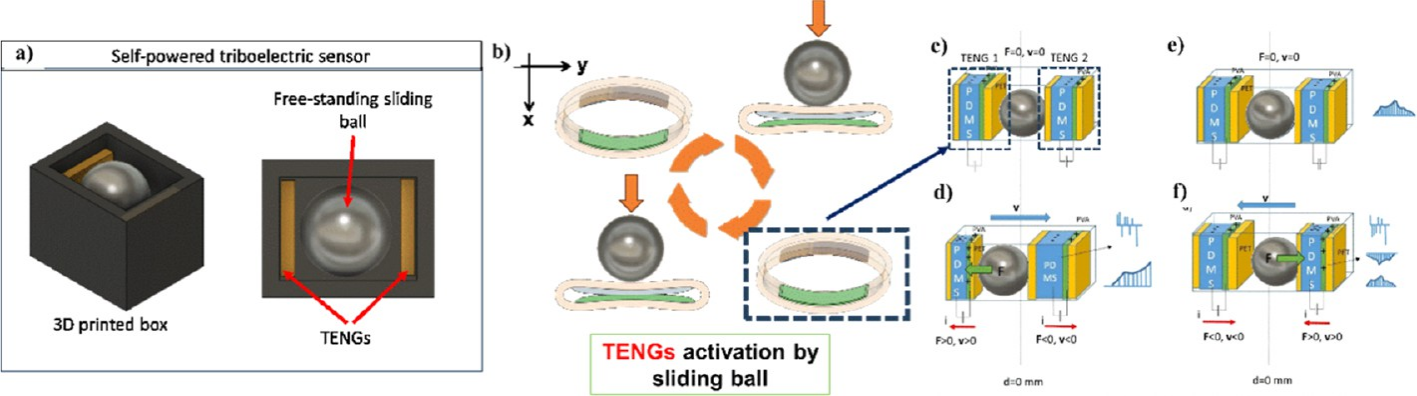
(a) Setup of 2D-SEISTENG. (b) 2D-SEISTENG transducer triboelectric layers and (c) 2D-SEISTENG operation when it is not moving (*F* = 0 and velocity *v* = 0, meaning the sensor is in a static mode or the metal sphere does not touch the tribo-layers). Reprinted with permission from.^5^ Copyright 2005, Elsevier; (d) the sensor moves to the right and the left TENG generates the positive component of the pulse under compression (the right one fulfils the negative part under relaxation for after remaining static). Reprinted with permission from.^5^ Copyright 2005, Elsevier. (e) Again, the ground does not move or there is no force supported by the tribo-layers. Reprinted with permission from.^5^ Copyright 2005, Elsevier. (f) When the ground or vibrating table moves to the left (a right TENG positive pulse is generated, repeating the cycle). Reprinted with permission from.^5^ Copyright 2005, Elsevier.

### 3.2 The Effect of the Electrical Induction in the Teng

It has been observed that an electric current is generated when there is no contact between the inertial mass and TENG layers. The PLA 3D printed prism box with two TENGs, one glued to the back and the other to the front, was screwed to a V10 shock table, which vibrated for different frequencies programmed with a PA100 amplifier controller. An inertial mass (a metal sphere or a cylinder), charged due to friction with the base of the box, slid on the bottom surface and hit the TENG sensors, which were compressed when it was in contact with them. Furthermore, a Quanser vibration table was also used for studying the same effect under different frequencies, elongations, and forces. As reported above, during the distance run by the inertial mass, a drift signal due to electric induction could be measured the same as the high pulse generated when impact with the TENGs was produced (Figure 4).

**Figure 4:**
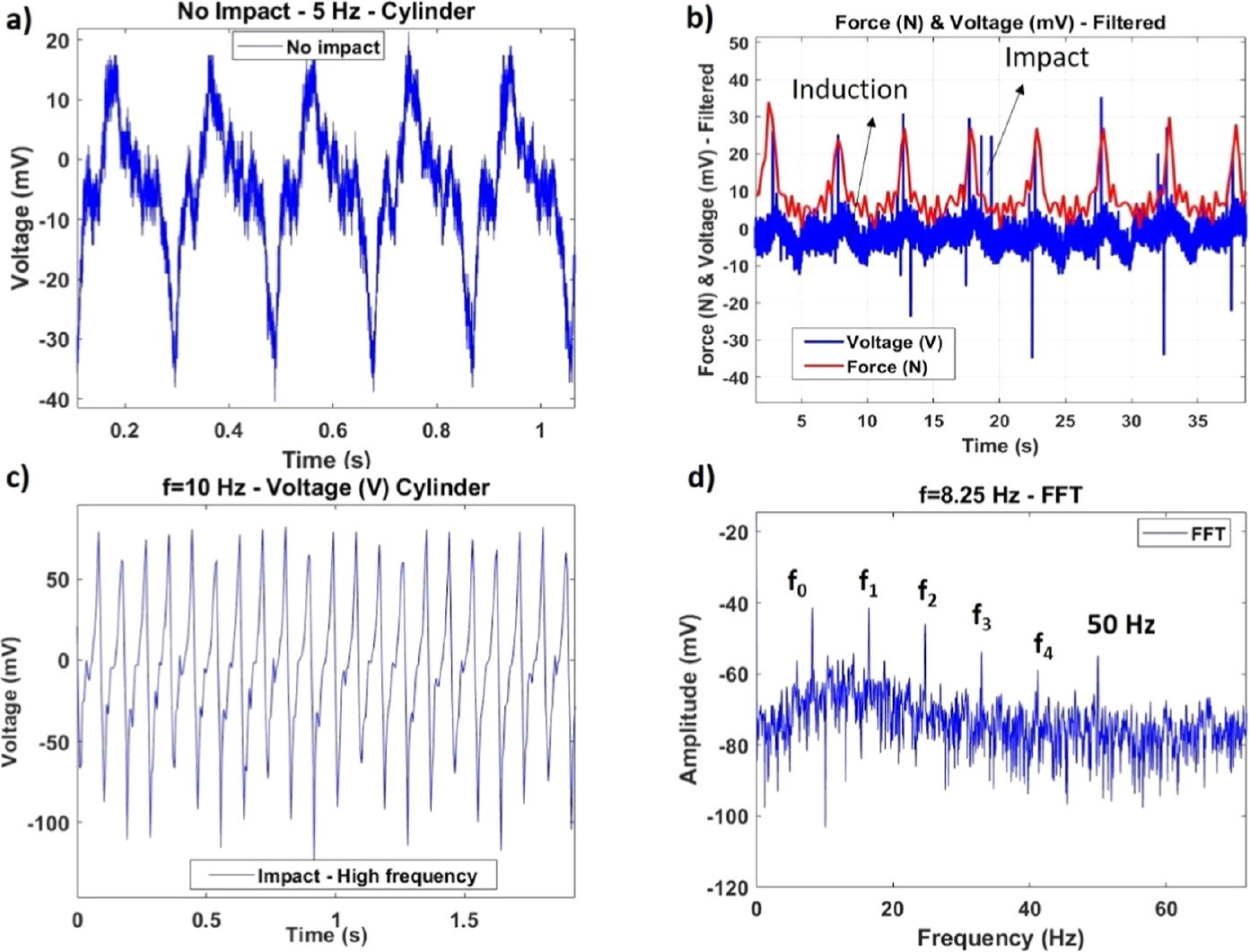
(a) Voltage generated by the TENG when a metal cylinder hit their layers due to the vibration of a shock table at 5 Hz when the TENG was under preload and no impact was produced. (b) Voltage and force values with a mix of impact and pure compression/traction modes but in this case with V signals generated by the TENG under traction/compression forces of *F* = 25–30 N.^5^ Reprinted with permission from.^5^ Copyright 2005, Elsevier. (c) Voltage generated by the impacts produced by the cylinder hutting the TENG at a 10 Hz frequency. (d) Fast Fourier transform (FFT) of the voltage generated by the TENG for an 8.25 Hz frequency with its subsequent harmonics.

Induction could be measured when the metal cylinder was sliding in the box base, which had been screwed to the vibration table (see Figure 4). Here, voltage generated by the TENG for pure induction (when the inertial mass is not in contact with the TENG) and a mix of impacts and induction phenomena caused by the metal cylinder are shown in Figure 4a for the excitation frequency of 5 Hz and in Figure 4b, where a mix of impact and pure compression/traction is shown. For this latter case, V signals were generated by the TENG under traction/compression forces of *F* = 25–30 N with the INSTROM testing mechanical machine. In Figure 4c, the impact of the metal cylinder on the TENG for a shaker excitation frequency of 10 Hz can be seen and in Figure 4d the FFT of the voltage generated for a frequency of 8.25 Hz is presented. Different harmonics, including the principal one, are shown (8.25 Hz, 16.5 Hz, 24.75 Hz, 33 Hz, 41.25 Hz, 49.50 Hz), meaning that TENG may also be used as a highly accurate high-frequency detector. The last harmonic of 49.5 Hz is mixed with that measured from the electric network (50 Hz), with this measurement (8.25 Hz) being performed with the metal sphere. For all these calculations, a Matlab program programmed by the authors with α = 0.01 was used to measure this induction effect (see Materials Section) and another one allowed obtaining the different frequency spectra by using the FFT algorithm.

### 3.3 Validation of 2D-SEISTENG as a High-Performance Frequency Acquisition System

To corroborate 2D-SEISTENG as an excellent frequency acquisition system, 1–5 Hz vibration table frequencies were used, with the sphere inertial mass hitting the two TENGs, each of them positioned one in front of the other and fixed on each of the front walls. Results are presented. As can be seen in Figure 5, where impacts are generated as the mass contacts the sensors, TENG pulses are well formed even for low frequencies. The pulses plotted either for the dynamic motion (Figure 5a,b,d,e,g,h) or in the Fourier space (Figure 5c,f,i) present a much better waveform than when using the system with an initial preload. However, in this last case, slower motion can be monitored.

**Figure 5:**
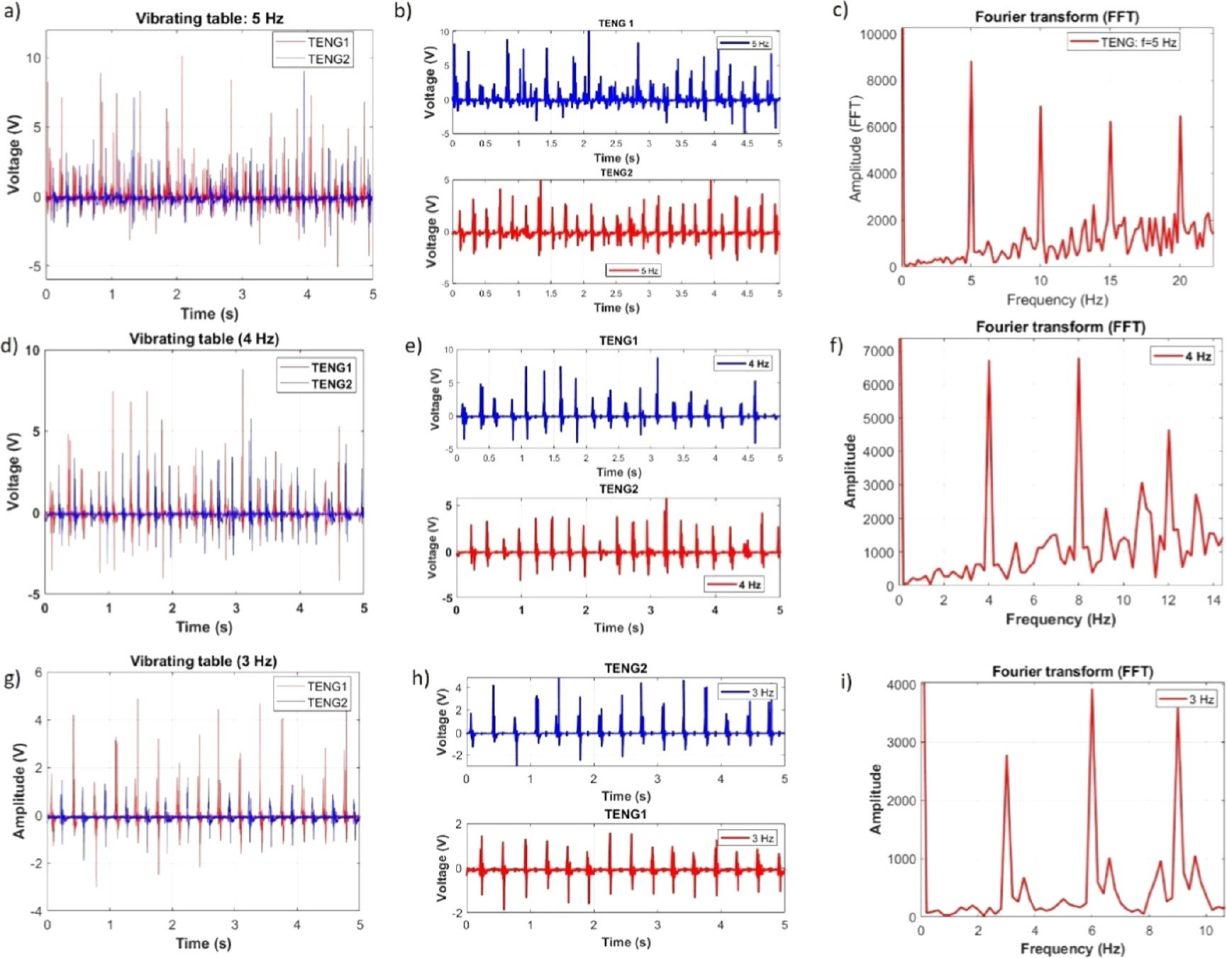
TENG pulses generated by the inertial mass of the seismic sensor device when there is not any preload for: (a) pulses together at 5 Hz; (b) separated pulses for 5 Hz; (c) FFT for 5 Hz; (d) pulses together for 4 Hz; (e) separated pulses at 4 Hz; (f) FFT for 4 Hz. Reprinted with permission from.^5^ Copyright 2005, Elsevier; (g) pulses together at 3 Hz. (h) Separated pulses for 3 Hz; (i) FFT for 3 Hz. Reprinted with permission from.^5^ Copyright 2005, Elsevier. TENGs did not have any initial preload by the inertial mass in the box.

The main concern of using this last configuration of impacts is that it is not as easy to study slow motion and fast events together unless either a high induction is generated or a thick TENG layer is slowly deformed. In addition, 2 TENGs (each on the opposite walls) enable the study to detect twice the frequency programmed for the vibrating table. However, FFT analysis was performed for only one of the sensors, obtaining the same frequencies as those programmed in the vibrating table.

### 3.4 Validation of 2D-SEISTENG as a High-Performance Velocity and Acceleration Acquisition System

Accurate D220 piezoelectric sensor voltage responses with outstanding waveforms were obtained for jaw velocities of 0.5, 1, 10, and 40 mm/min for a force of 1 N exerted on the piezoelectric sensor (see Figure S3a). It was checked that variations of such force for the same velocity did not change the output signal, either in the waveform or in the amplitude response. It is clear that D220-A4BR-1305YB provides a very low-noise signal. The SNR (signal-to-noise ratio) for the D220-A4BR-1305YB is 10 mV in static conditions, while for the TENG layers, it is 150 mV, and when the vibrating table is working, the SNR is three times higher for both cases. A comparison between the D220-A4BR-1305YB piezoelectric sensor and TENG seismic sensor is presented in Table S4 and Section S1. As informed in this table, one of the most outstanding characteristics of the 2D-SEISTENG transducer is the versatility of its electrical/mechanical characteristics because although in this study, the TENG is made of PDMS/PVA, in general, it can be fabricated with any other triboelectric material with its intrinsic properties. Often, they are made of polymer composites; however, they could be hard and made of other materials, even semiconductors. In addition, the TENG sensitivity is dependent on the voltage probe used to measure the signal. Furthermore, a calibration of the voltage generated by the TENG and the voltage generated by the piezoelectric sensor was performed for the same excitation frequencies, resulting in a linear calibration fit (Figure S3b). In addition, a linear calibration curve of the jaw velocity (Figure S4a) and acceleration (Figure S4b) that are the same as the ones supported by the TENG layers was obtained for the same force supported.

Table S4 highlights the sensitivities and dynamic range of both TENG and piezoelectric sensors. In order to calculate TENG deviation sensitivity due to temperature (T), we have mechanically excited the TENG with a dynamic mechanical analyzer (DMA, TA Q800) working at 2000 μm of separating distance and 10 Hz as it has been reported in previous works^60^ (see Figure S11). In addition, we have evaluated the output voltage response of the TENG for different values of humidity (Figure S12) under the same procedure as in.^60^ As reported above, TENG sensitivity for different humidity values is 1.8 (0.18 V/%) and for different temperatures 0.014 V/°C (1.5%/°C). Regarding the piezoelectric sensor, the temperature sensitivity is of 0.03 Vm/N that is the same as 3%/°C. We have included the sensitivity relation between TENG and PIEZO, according to the plot of Figure S3 b (S_piezo_ = 10.65xSTENG −0.55). In addition, other parameters such as the calculation of the deviation from linearity is explained in Section S3.

Furthermore, a comparison of sensitivities between the different seismic sensors that are sold in the market is presented in Table S5. Here, geophones, seismometers, hydrophones, MEMs, velocity, and acceleration sensors are depicted and different sensor characteristics (intrinsic sensitivity, natural working frequency, distortion, operation position, temperature range operation, operating humidity, pressure, portability, noise, and the use of power supply) are compared.

### 3.5 Simulation of the V Generated for Different Compression Forces

A simulation model, such as the one described in ref,^5^ has been evaluated where the basic equation for the contact-mode TENG was employed to observe the influence of the voltage under different compression forces. In our case, it was solved when the TENG was under a preload. It can be observed that the higher the force applied when layers are in contact, the higher the voltage achieved. In addition, the fit is not linear, and there is a limit force for which voltage is saturated. In Figure S8a, force vs voltage generated by PDMS–PVA-based TENG is simulated. In this simulation, the first layer (PDMS) thickness is 4 mm, and the second one (PVA) is only 0.5 mm. The maximum elongation supported is of 3.6 mm from when they are in contact, meaning that compression begins with a preload and layers (mainly PDMS) reduce their thickness in a value of such elongation. In Figure S8b, a simulation of the voltage generated by the mechanical jaws in compression/relaxation mode for a force near to the maximum saturated voltage value is presented.

### 3.6 Electrical Characterization of 2D-SEISTENG

Voltage and current generated by 2D-SEISTENG vs frequency of the vibrating table are presented in Figure S5. It can be seen that the higher the frequency applied, the higher voltage and current amplitude of the TENG is generated. In addition, with the same previous 2D-SEISTENG setup, different current, voltage, velocity, and acceleration values were plotted for the frequencies of 1 and 5 Hz (see Figure S6) and the same magnitudes of current and voltage but for different resistance loads (as shown in Figure S7). Plots of voltage and current vs force were obtained by using the force values associated with voltage vs acceleration curves shown in Figure S4b and multiplying them by the mass. In addition, velocity and acceleration were obtained from the calibrations shown in Figure S4a,b. Regarding Figure S8, simulated plots of voltage and current vs time were obtained by using Python in Google Collaborate as reported in.^51^ According to Figure S7, it can be concluded that the highest power value is achieved for 10^5^ Ω resulting in a maximum electrical power of 9 μW. The electrical power generation curve is shown in Figure S9. In addition, the relation between Voc and Isc of the TENG seismic sensor was also obtained (see Figure S13). With this relation, the Isc vs temperature and humidity was also calculated (Figures S11 and S12, respectively). TENG temperature response was obtained with the DMA and a controlled temperature chamber positioned on the plates that allowed modifying the temperature between 30 and 90 °C. Furthermore, TENG was exposed to different humidity values programmed in a climatic chamber and after voltage response was measured (see Figure S12) for room, 70% and 90% humidity conditions. In addition, the dynamic range (DR) of both seismic sensors was also calculated and very similar values (79 dB) were obtained (see Table S4).

### 3.7 TENG Voltage Signal Remotely Transmitted to the Cloud

In order to acquire and transmit the TENG voltage signal to the cloud, the STEMLab 125–14 EDU PACK^61^ was used as shown in Figure 6a. This compact electronic board was deployed to remote monitoring on the internet TENG voltage signals through a LabVIEW interface Figure 6b,c. These signals were transmitted via Wi-Fi and locally generated by a TENG, which was hand tapped at different frequencies and was connected with its electrodes to the STEMLab board. Furthermore, rectangular and triangular waveforms generated with a function generator at frequencies from 0.5 to 30 Hz were also locally and remotely monitored, and in all the cases, no information package was lost when visualizing such signals on the internet. The high reproducibility of the local measurements in IoT platforms is shown in Figure 6d–f for TENG and function generator signals of frequencies 1 Hz, 6 Hz, 10 Hz, 100 Hz, and TENG raw signals for two experiments performed via finger tapping with a TENG, respectively (see Supporting Information Video SV1). To evaluate wireless performance, a latency test was conducted using Internet Control Message Protocol (ICMP) echo requests over WiFi. Results showed no packet loss and round-trip times between 4 and 7 ms, confirming a stable connection suitable for data acquisition. The use of the electronic board described helps users to read locally earthquake signals generated by a TENG when vibrating artificially (or not) in the ground from anywhere and at any time with always a device with an Internet connection.

**Figure 6:**
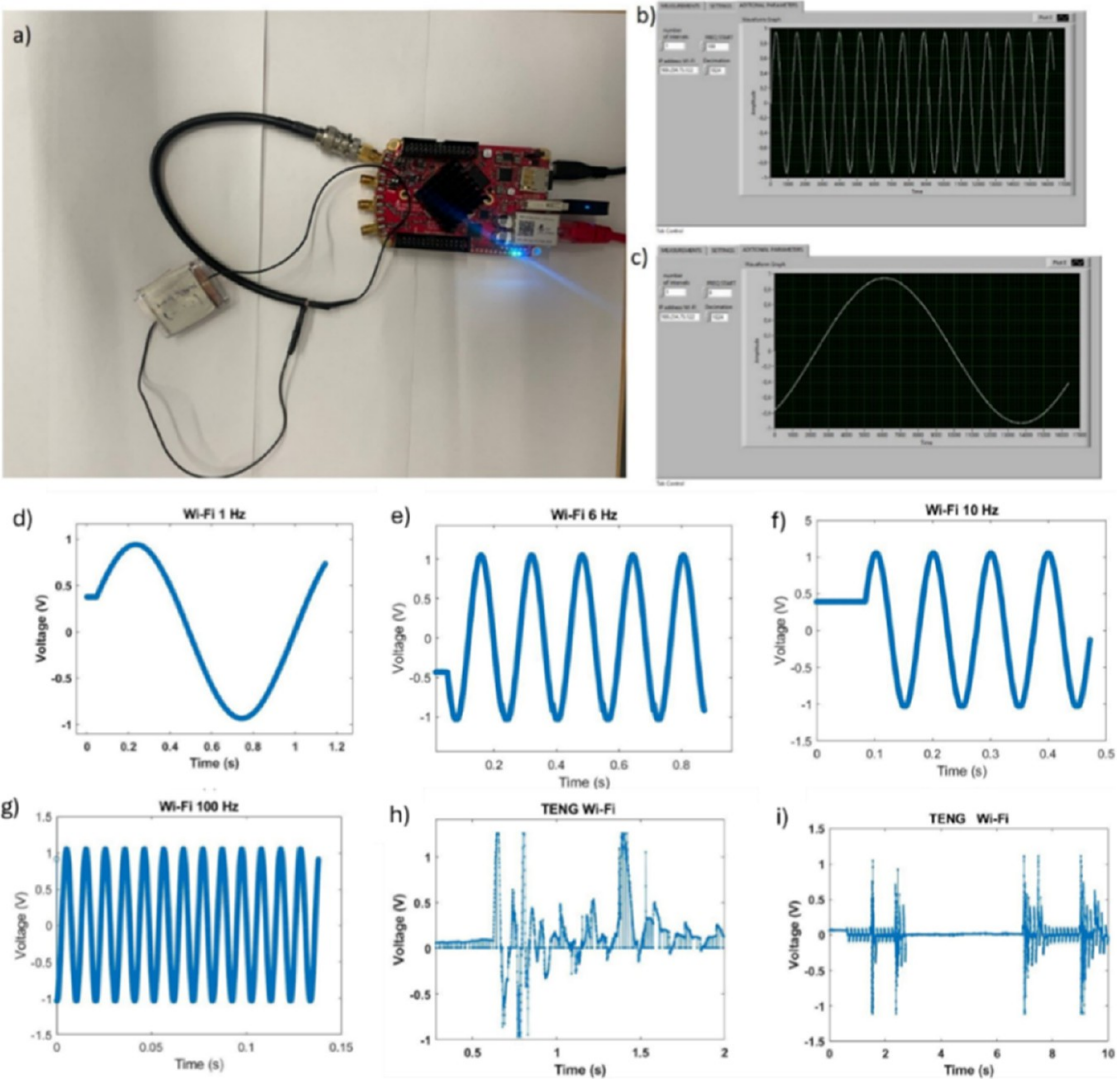
(a) STEMLab 125–14 EDU PACK with the TENG coupled; (b) LabVIEW visual programming environment used to monitor signals sent via Wi-Fi and received for *f* = 13 Hz; (c) for *f* = 1 Hz, Wi-Fi reception voltage signals generated with a frequency generator and emitted by the STEMLAB 125–14 EDU PACK with frequencies of (d) 4 Hz, (e) 6 Hz, (f) 10 Hz, and (g) 100 Hz and generated by a TENG via finger-tapping, magnified (h) and in normal scale (i), all of them visualized on the internet via http.

### 3.8 TENG Stability and Transmission Capability of the TENG Seismic Sensor

Since the long-term stability of TENGs must be evaluated, we performed this assessment again but using a different approach. As previously described in ref,^60^ we measured the stability of the device with a DMA (TA Q800). The oscillation frequency was set to 10 Hz (see Figure S14), and the amplitude exhibited only a minor decrease of about 1% by the 500th cycle and remained essentially unchanged thereafter. Furthermore, the seismic remote signal sent by the STEMLab 125–14 EDU PACK (Red Pitaya) did not show any transmission signal loss during the tests. This means that the seismic sensor stability is high for long time periods (>1000 s), and it is the same as the TENG transducer meaning that the deployment of multiple sensor nodes in real-world environments is a valuable direction for future research.

Despite its transducer, the system has demonstrated excellent temporal stability in a continuous acquisition scenario. For instance, when subjected to a constant +1 V input signal over extended periods (*t* = 5000 s), the acquisition system maintained a measurement precision within the range of ±3500 μV. The accuracy of the acquisition system was further evaluated using a sinusoidal input signal with zero offset and a peak amplitude of ±0.5 V. The measured amplitude accuracy across 0–50 Hz frequencies exhibited a standard deviation of 12 mV, corresponding to an amplitude accuracy of 97.1%. Similarly, the sensor connected to the electronic board exhibited a standard deviation of 0.082 V under fixed connection, further supporting its signal reproducibility over time. In addition, the system’s ability to down sample signals using built-in decimation allows it to handle low-frequency seismic signals without compromising accuracy. A collaborative operation of multiple sensors represents an important performance for our future research.

In the current study, the experimental tests were conducted using a single electronic platform with a dual-channel ADC system for simultaneous high-resolution acquisition of two TENGS’ input signals. It is featured by the AD9649 ADC sharing a common clock, ensuring synchronized samples from both channels. This setup allowed us to validate the acquisition, buffering, transmission, and visualization stages within a controlled environment while simulating two simultaneous TENGS sensors’ operation on a single node. In our implementation, a buffering mechanism and TCP-based communication with a 500 ms timeout were used to mitigate potential issues related to Wi-Fi latency and asynchronous data transfer. Signal acquisition was initiated via SCPI commands using a LabVIEW-based visual programming interface.

### 3.9 Simulation of a Real Earthquake (Kobe) and Detection Signal

A simulation of the Kobe earthquake,^62^ also known as the Great Hanshin earthquake, was employed for testing our device. This ground motion was an event that occurred on January 17, 1995 in the Kansai region of Japan, having a Richter-scale magnitude of 6.9. First, the vibrating signals were programmed with the MATEST software and detected by an accelerometer incorporated into the table. The waveform and amplitude of the oscillations generated by the shocker in the time domain and measured by the accelerometer integrated can be seen in Figure 7a. The signals generated by the TENG and measured with the Picoscope DAQ are shown in Figure 7b. Furthermore, this last signal, but magnified, is shown in Figure 7c. Later, the seismic TENG signal was smoothed by using a MATLAB program developed by the authors and the MATLAB toolbox. A comparison between this filtered signal generated by the TENG caused by the spherical inertial mass impacting on its layers and that measured by the vibrating accelerator joint to the table is shown in Figure 7d, reaching a high degree of similarity (75%).

**Figure 7:**
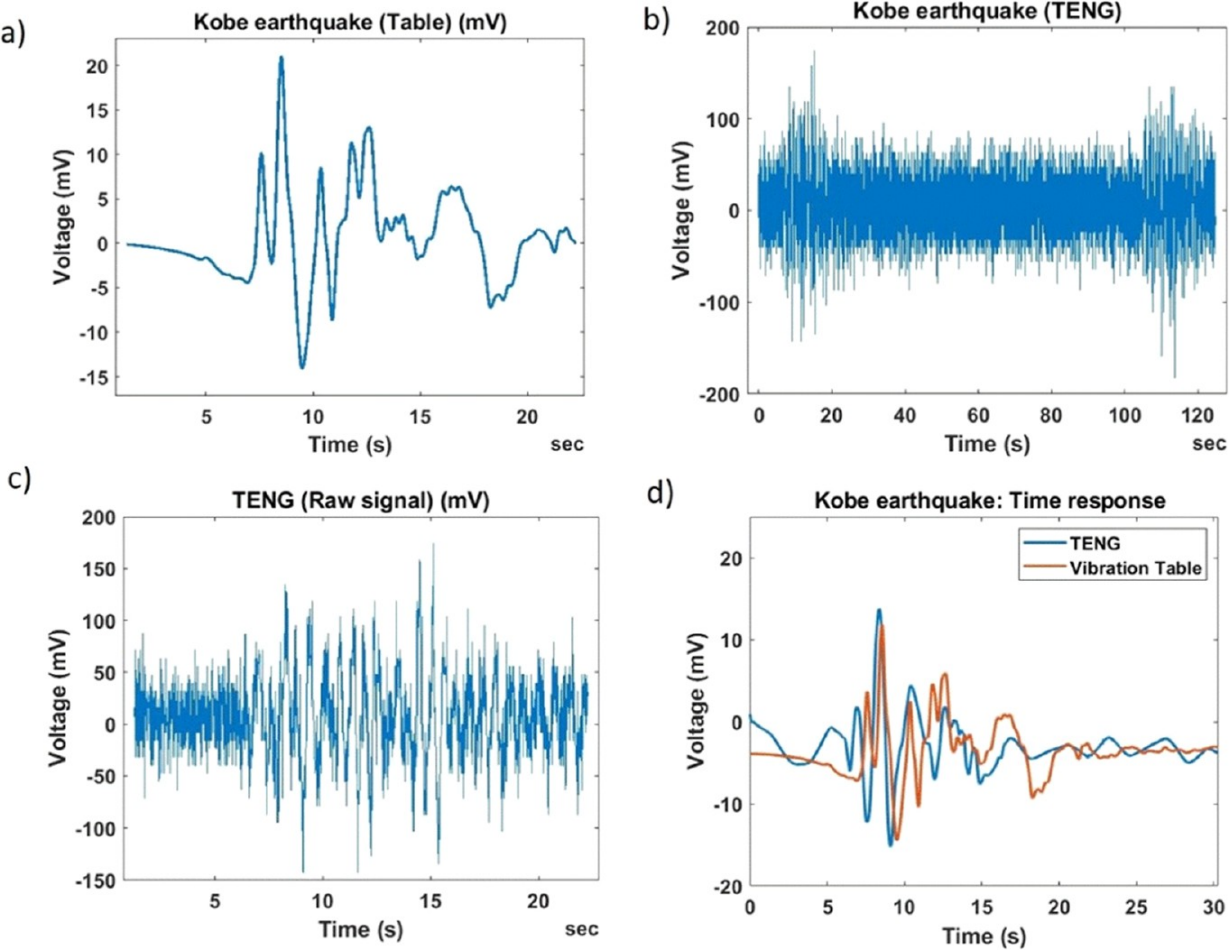
(a) Displacement (V)–time (s) graph from the Kobe earthquake with a maximum displacement of 0.17 m programmed in the vibration table and measured with the table accelerometer. (b) The same as in (a) but measured with the TENG, with two vibrating shocks. (c) The same as in (b) but signal magnified in the time scale for the first shock. (d) Both time vibration table accelerometer and TENG signals to see their similarity, with TENG signal previously smoothed.

The same results as in Figure 7, though in the frequency domain, are presented in Figure 8. Again, the MATLAB signal analysis toolbox was used and FFT was applied to the accelerometer and TENG voltage signals. Raw signals measured by the accelerometer and by the TENG in the frequency domain are shown in Figure 8a,b once the FFT was applied to such a time signal domain. In order to compare the signals, low band-pass filtering with a cutoff frequency of 10 mHz and a smoothing function were applied to the raw time signals. As shown in Figure 8b, frequencies were systematically shifted to the right part of the spectrum (Δ*f* = 12 Hz) due to mainly the smooth parameters and low band filters used such as the moving mean filter that attenuate high-frequency components and preserve low-frequency ones. Furthermore, frequency limits were between 1.23 and 25 Hz and the time window between 1.28 and 22.27 s was considered. After a smoothing factor of 0.25 was applied three times with the moving mean and after, the frequency spectrum was calculated and shown. In addition, the difference in the external signal and the one measured with 2D-SEISTENG is because of either the noise existing in the raw voltage generated by the TENG, the difficulty of the inertial mass to follow the oscillations of the vibrating table due to friction between the box and the mass or to electrical induction caused by the shocker. A comparison between those of the vibrating table and the TENG is presented in Figure 8c, where the frequency shift was subtracted in the TENG spectrum causing a displacement to the left and an overlap of both spectra with the lower-frequency peaks (0.25 and 0.5 Hz). The same spectra but magnified in the frequency domain are shown in Figure 8d.

**Figure 8:**
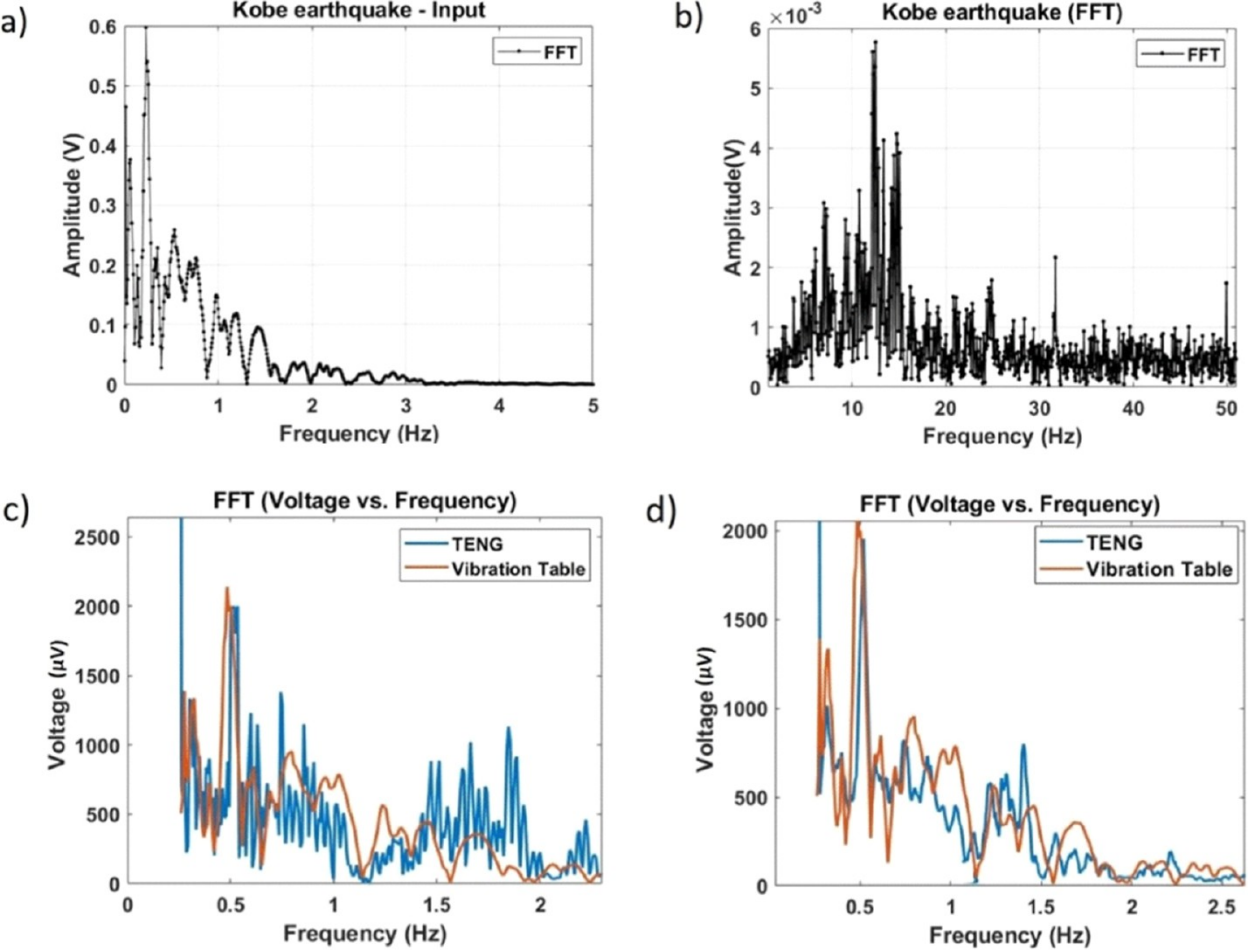
Amplitude of the first harmonic of the FFT for (a) the Kobe earthquake signal programmed and generated in the shocking table, (b) for the TENG voltage signal generated by the seismic inertial mass, (c) for both vibrating table accelerometer and TENG signals, this last one smoothed and scaled, and (d) for both too, but magnified and again smoothed and rescaled in frequency to make a better comparison.

The high degree of agreement between the vibrating table accelerometer and the TENG seismic sensor is shown in Figures 9 and 10, where either the power spectra with the same profile, the spectrogram with the same maxima in the power spectrum for the same frequency and time, or the scalogram with the same maxima in the continuous wavelet transform (CWT) for the same frequency and time demonstrates the potentiality of the TENG seismic sensor proposed.

**Figure 9:**
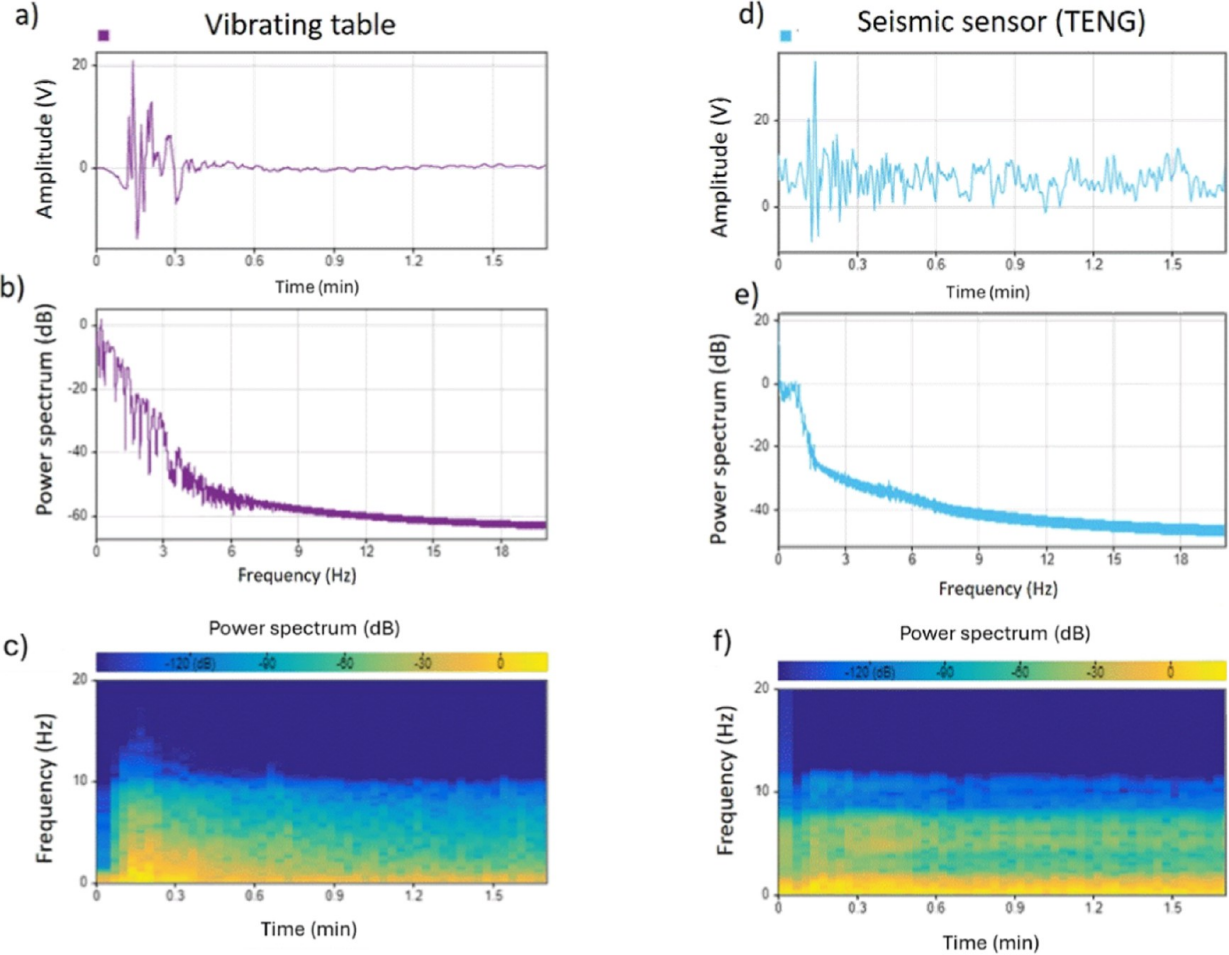
(a) Voltage measured by the vibrating table accelerometer. (b) Power spectrum (dB) vs frequency. (c) Spectrogram (frequency (Hz) vs time (s) vs power spectrum (dB). (d) Voltage measured by the seismic sensor TENG. (e) Power spectrum (dB) vs frequency measured by the TENG. (f) Spectrogram (frequency (Hz) vs time (s) vs power spectrum (dB) measured by the TENG.

**Figure 10:**
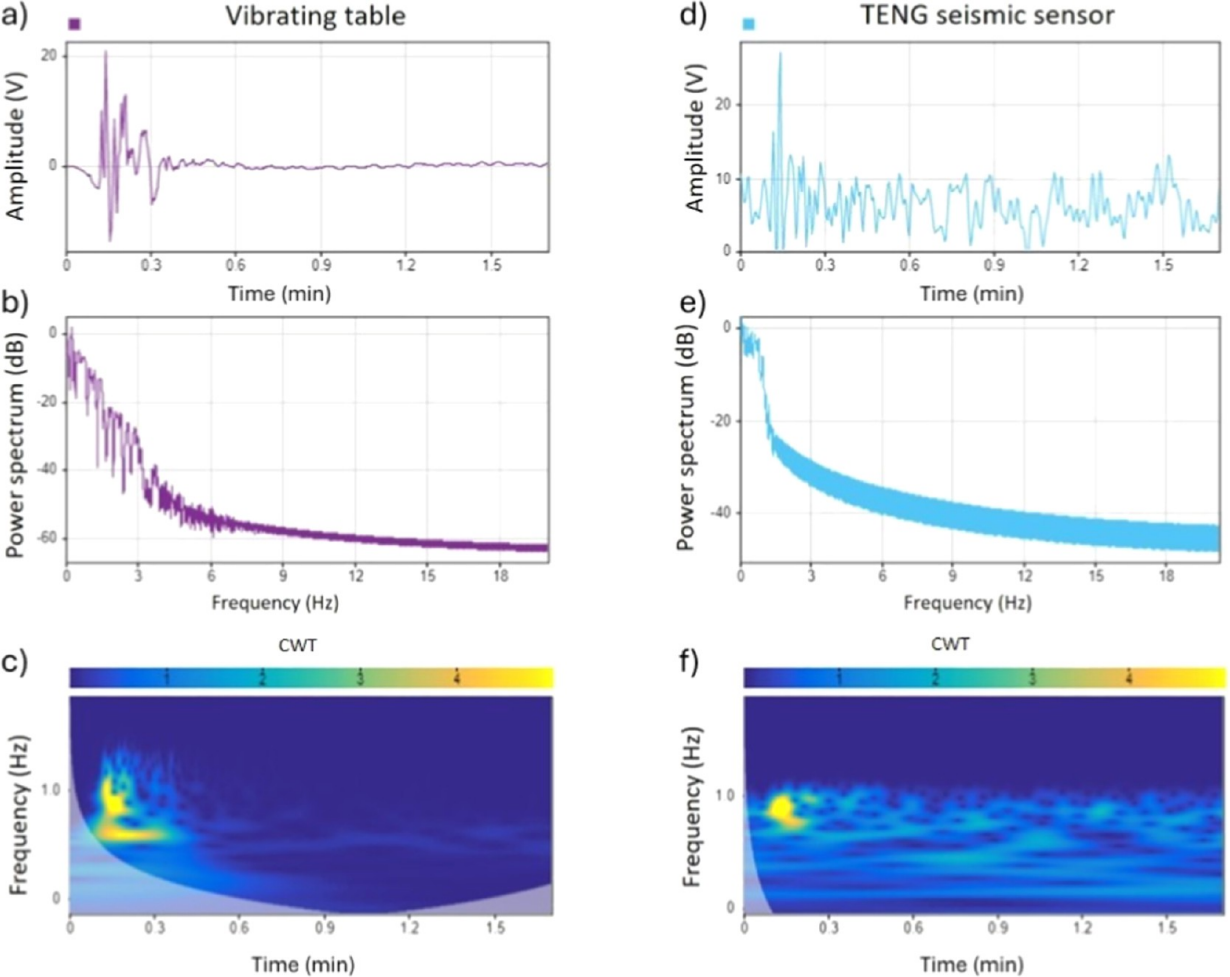
(a) Voltage measured by the vibrating table accelerometer; (b) power spectrum (dB) vs frequency; (c) scalogram (frequency (Hz) vs time (s) vs power spectrum (dB) vs continuous wavelet transform (CWT); (d) voltage measured by the seismic sensor TENG; (e) power spectrum (dB) vs frequency measured by the TENG; and (f) scalogram (frequency (Hz) vs time (s) vs continuous wavelet transform (CWT) measured by the TENG.

The first harmonic contains the majority of the vibrational energy; consequently, the noise level is considerably reduced in this spectral region. As a result, when low band-pass and smoothing filters are applied, the waveform and frequency content remain largely unchanged with respect to the unfiltered raw data. For this reason, the principal harmonics obtained from the FFT were used for subsequent analysis when detecting the excitation produced by the vibration table during the simulation of the Kobe earthquake.

In this simulated scenario, the discrepancies observed between the actual excitation and the signal recorded by the TENG seismic sensor can be attributed to several factors. One source of error is the noise generated when the triboelectric layers are extremely close to each other, just before the impacts corresponding to the seismic excitation begin, an effect that was previously reported. Additional deviations arise from the smoothing and filtering procedures applied to the TENG’s output. Another contributing factor is that TENGs do not behave as ideal springs; their elastic response is limited, particularly under high-impact vibrations, which reduce the fidelity of the mechanical model.

When comparing the vibration signals measured by the TENG with those obtained from the accelerometer, a high degree of agreement was observed between the waveform produced by the shocker and that detected by both devices, although some TENG-derived frequencies exhibited slight shifts. As previously mentioned, the closer the system operates to its resonant frequency, the more difficult it is for the inertial mass to accurately follow the ground motion.

It is also important to emphasize that, during ground vibration and translational movement, the inertial mass rolls on the flat surface without sliding. This implies that friction is generated at the interface. Under these conditions, the operating principle of the 2D-SEISTENG closely follows the 2D forced-friction damping model. According to Figure 10, high-amplitude signals in the frequency range of (0.8–1.2) Hz were observed after the sensors began to measure at 0.2 min. There is a high similarity of the dynamic signal (Figure 10a,a’), the power spectrum and its falling edge in the frequency range of (1–2) Hz (Figure 10b,b’), and the stalograms (Figure 10c,c’), where 3D plots of the variables such as frequency (Hz), time (min), and CWT are depicted for either the vibrating table accelerometer or the TENG seismic sensor (2D-SEISTENG). An explanation and the code used to calculate the power spectrum (dB) and the CWT using the seismic data are summarized in Section 4.

Considering the solutions given by the forced damping harmonic model given by eq 9 and 10 in Section 2.8 and in Figure S18 where these solutions are plotted with MATLAB, the relative displacement maintains an almost constant phase lag with respect to the angular acceleration of the platform, at an angle close to 180°, up to approximately 2 Hz. For higher frequencies, a significant change in the phase lag begins to be observed, indicating that the system stops behaving as an almost static relationship and enters a range where dynamic effects are dominant. The system’s resonance is at 2.64 Hz.

On the other hand, the magnitude starts to increase with frequency due to approaching the system’s resonance, causing the mass–spring–damper assembly to act as a mechanical amplifier, increasing the amplitude of the relative displacement in response to the base excitations. In the frequency range from 1 to 2.64 Hz, this increase becomes significant; for this reason, the measurements taken with the SEISTENG show peaks of higher amplitude than those recorded by the used accelerometer as reference (see Figure S19c,d with the experimental results obtained with the Kobe simulated earthquake).

It is clear that the best seismic setup configuration to measure ground motion is such that the inertial mass remains in its original position on the box floor without moving far away from the resonance (ω≫ω_0_) or (ω≪ω_0_). In the situation ω≫ω_0_, it will be only the box with the TENG that it will move causing impact with the inertial mass that is in a static position, and when ω≪ω_0_, the mass will follow the base motion. This is the case for the other experimental test we have performed with the vibrating table of CEDEX, where Lorca earthquake, with a much lower intensity than Kobe’s (TENGs are more similar to springs) and with a resonant frequency of 6.1 Hz, was simulated (see the next section). The explanation of its better performance due to the earthquake lower intensity is the following: there are fewer rebounds between the inertial mass and the box walls as the TENG transducers work as springs accomplishing the force damped oscillator model. It is in this situation when inertial mass follows the ground motion and the base (box) more easily. The optimum performance will be when TENGs behave as springs, accomplishing the forced damping elastic model. The next configuration of SEISTENG will make use of TENGs fixed to springs placed on the walls.

Furthermore, acceleration and velocity of the seismic waves simulated with the vibration table were compared either measured with the piezoelectric commercial sensor or the 2D-SEISTENG, as shown in Figure 11. This signal was determined according to the calibration curves performed with the TENG and the piezoelectric sensor, which are shown in Figures S1, S3a and S4a,b. As observed, a great similarity of the signal between the acceleration and velocity obtained with the piezoelectric (vibrating table) and the TENG was achieved, which further shows that the 2D-SEISTENG can work as a velocity and acceleration sensor.

**Figure 11:**
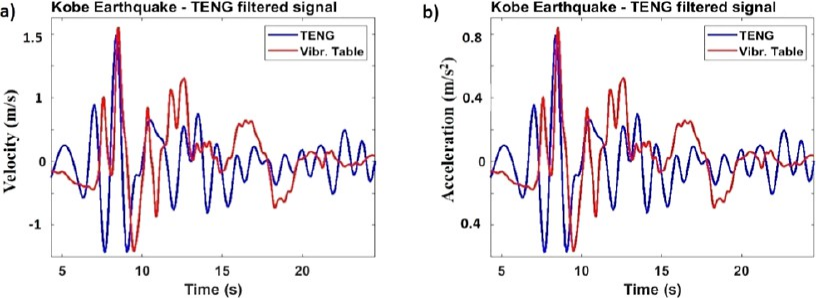
(a) Velocity (m/s) and (b) acceleration (m/s^2^) of the Kobe earthquake generated with the vibrating table.

### 3.10 Simulation of a Real Earthquake with Lower Magnitude (Lorca) and 2D-SEISTENG Signal Detection

Two 2D-SEISTENGs and one 1D-SEISTENG used to detect vibrations in the HNE, HNS, and HNZ, respectively, were fixed to the triaxial vibration table of CEDEX. These three parts of SEISTENG will conform later to the 3D-SEISTENG, described in the next section. The seism produced was the same as the one that took place in 2011 and was much lower in intensity than the one in Kobe. This lower intensity allowed SEISTENG to operate with a better performance than with the high-impact Kobe simulated one as the inertial mass could follow the ground motion with high accuracy.

The Lorca earthquake was a moderate 5.1 Mw (magnitude) earthquake that occurred at 6:47 p.m. CEST (16:47 UTC) on May 11, 2011, near the town of Lorca, causing significant localized damage in the region of Murcia, Spain.^63^ In Figure 12, results obtained with 2D-SEISTENG are summarized. As shown in this figure, the two quakes simulated in the vibration table could be detected (Figure 12a) by the TENG transducer. In order to determine the dominant frequencies of these two Lorca quakes, the FFT of the signal was plotted either in linear or in logarithmic scales (Figure 12b,c), respectively). To finish, dominant frequencies were marked in the plot (Figure 12d), and they are shown in Table S6.

**Figure 12:**
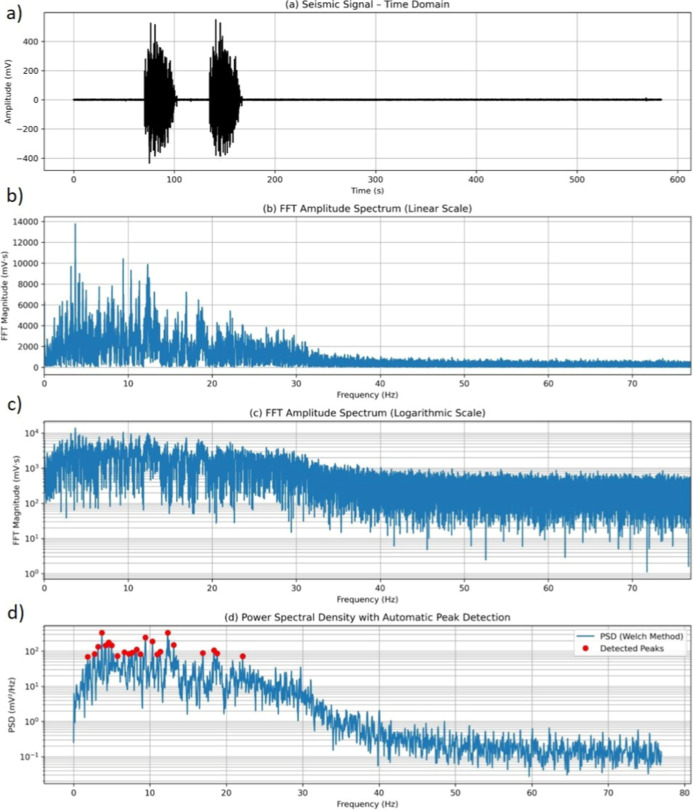
(a) Real simulated Lorca earthquake signals measured with 2D-SEISTENG. (b) Fast Fourier transform (FFT) amplitude spectrum. (c) Logarithmic scale of b). (d) Power spectral density with automatic peak detection.

### 3.11 The 3D-SEISTENG Novel Design

Making use of the 2D-SEISTENG prototype, together with the 1D-SEISTENG model described by the authors,^4^ 3D-SEISTENG was designed with Freecad^64^ and fabricated with a 1 header BAMBOO 3D printer. This design is shown in Figure 13 and has two prism boxes in perpendicular directions (2D-SEISTENG), extending in the *X*–*Y* plane. A third part, constructed along the third *Z* axis, is made of an empty cube where the TENG is resting on its base (see Figure 13a,b,d,e). Tribolayers were compressed with a weight of 0.5 kg placed on their surfaces. Furthermore, a photo of the fabricated box with a LabVIEW interface programmed for the motion detection in the 3 directions of the space is shown in Figure 13c). The signal produced by the tribolayers in the three directions of the space can be seen in video SV2 of the Supporting Information. TENG signals were monitored with a National Instruments data acquisition system (DAQ) (NI-3001) by making use of three channels corresponding to each of the vibrating directions. In Figure 13d,e, 3D-SEISTENG.stl files are shown in solid and wire view and the two X-channels, the only one Y-channel and the Z-channel is shown.

**Figure 13:**
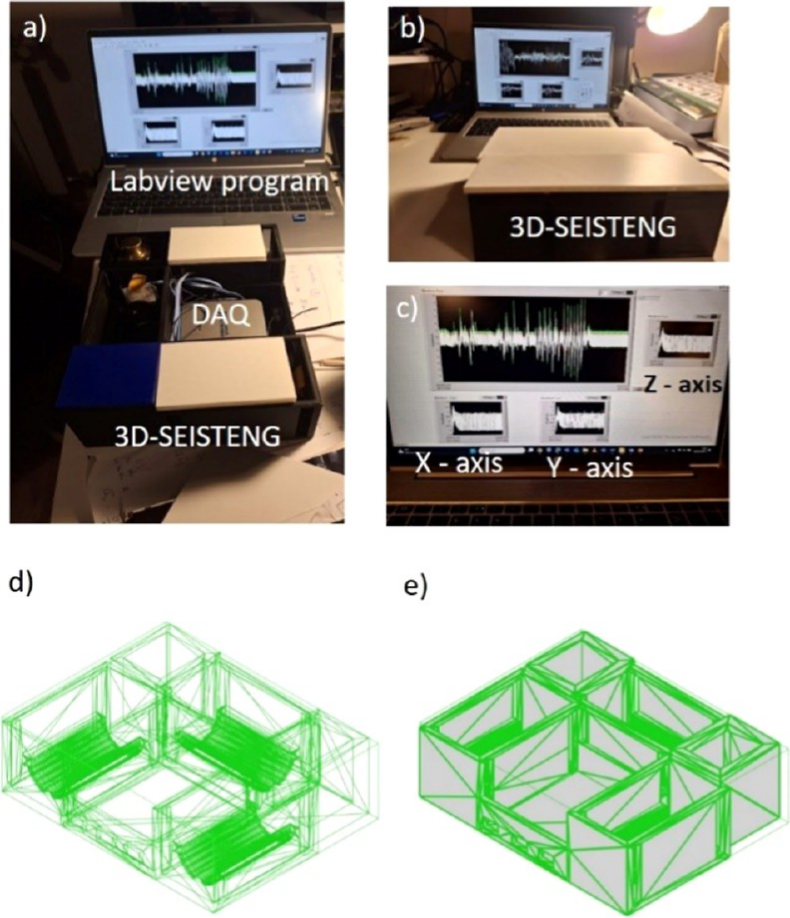
(a) 3D-SEISTENG components and LabVIEW program; (b) general view of 3D-SESITENG and setup; (c) 3D-SEISTENG LabVIEW interface; (d) 3D-SEISTENG case with the STL file with a wire structure. (e) 3D-SEISTENG solid case.

As reported above, 3D-SEISTENG consists of three fundamental parts associated with the motion directions, the HNE, the HNS, and the HNZ components. Furthermore, HNE and HNS correspond to the plane motion, and as a result, they are the 2D-SEISTENG modules. Conversely, HNZ corresponds to 1D-SEISTENG.^4^ All these three parts were fixed on the triaxial vibrating table where Lorca earthquake was simulated (see video SV3 of Supporting Information). As shown in Figure 14a, only two quakes were generated in the first experiment. Horizontal-to-vertical spectral ratio (HSVR) was calculated and allowed determining the fundamental frequency of the site that is 6.09 Hz (Figure 14b,c).This means that the ground at this site naturally resonates at ∼6.1 Hz, and seismic waves near this frequency are preferentially amplified by the local soil structure. The experiment will allow estimating the soft layer thickness that is likely in the order of 10–25 m, being the typical shear velocities in the range of 300–600 m/s. In addition, another longer simulation test of more than 3000 s with 14 quakes was detected by 3D-SEISTENG (Figure 14d). Again, horizontal-to-vertical spectral ratio (HSVR) was calculated (Figure 14e), and after being smoothed (Figure 14f), the fundamental frequency of the site was calculated. The HVSR analysis indicates a fundamental site frequency of approximately 11.4 Hz, suggesting very shallow stiff soil or weathered rock conditions. Using a 1D resonance approximation, the thickness of the surficial layer is estimated to be on the order of 10–15 m for plausible shear-wave velocities between 400 and 700 m/s. According to Eurocode 8,^65^ the site can be classified as class B, corresponding to stiff soil or weathered rock. However, if we consider the other lower frequencies (that includes the one of 6.1 Hz), the HVSR analysis reveals fundamental site frequencies ranging from approximately 6.1 to 11.4 Hz across the study area. These values indicate shallow stiff soil to weathered rock conditions, with estimated sediment thicknesses between 8 and 25 m. The observed spatial variability in fundamental frequency suggests lateral heterogeneity in the near-surface structure, likely controlled by variations in sediment thickness or bedrock depth. According to Eurocode 8, again, the sites can be classified as class B (stiff soil and weathered rock).

**Figure 14:**
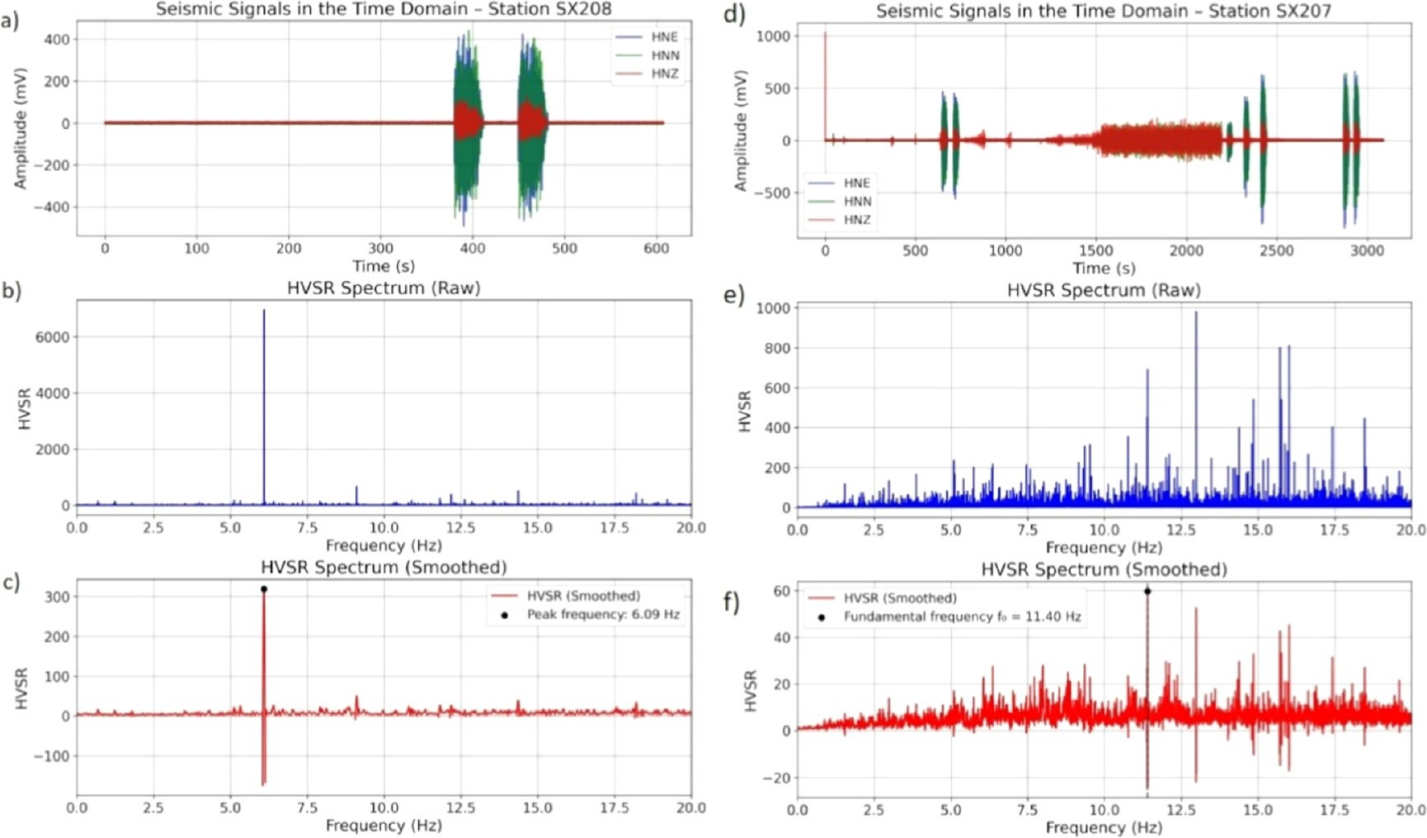
(a,d): Real signals measured in the time domain with 3D-SEISTENG modules. (b,e) HSVR spectra of the oscillations produced. (c,f) Smoothed HSVR spectra.

## 4 Conclusions

This work presents a unique seismic sensor based on the triboelectric energy that is able to detect ground motion. A seismic prototype was fabricated with a 3D printer that consisted of an inertial mass inside a prism box with a TENG fixed to the front walls so that it could receive the impact of the inertial mass (a metal sphere). Two realistic seisms (the first from the Kobe earthquake and the second from the Lorca earthquake) were generated, each of them with different resonant frequencies and with TENG signals reproducing with high accuracy the excitation energy generated by a vibration table, either in the time or the frequency domain. The configuration of the prototype (2D-SEISTENG) allowed measuring in the *X*–*Y* plane and together with the last work of the authors, in the 3 directions. In addition, a 3D-SEISTENG prototype was designed and fabricated for the next works, and each of the modules was tested separately in a triaxial vibrating table owned by CEDEX for the same simulated earthquake as the one that took place in 2011 in Lorca, Murcia, Spain. Some analysis of the signals obtained allowed understanding of the physical characteristics of the earthquake, the same as the characteristics of the soil. These results demonstrate the capability of TENG seismic sensors and, over more, 3D-SEISTENG, to be used as low-cost and excellent detectors in any kind of ground motion. Furthermore, such low cost—together with IoT communication protocols integrated in the electronic system—will allow the creation of a novel global network of this kind of seismic sensor that will generate data for either personal or public use in general that will be stored in the cloud and enable it to be used worldwide.

## Supplementary Material



## Notes

Calculation of the TENG elastic constant; different figures related to the experimental setup; calibration curves with the TENGs; simulations of the Isc and Voc performed with Python in Google Collaborate and electrical measurements; also, a comparison table of the D220-A4BR-1305YB piezoelectric sensor and 2D-SEISTENG transducer; seismic vs piezoelectric sensor sensitivities and dynamic range the same as velocity and acceleration physical magnitudes; in addition, different sensor characteristics sold in the market from different companies; dominant frequencies and PSD measured with 2D-SEISTENG of the Lorca earthquake simulated in the CEDEX (Centro de Estudios y Experimentación de Obras Públicas) vibrating table; furthermore, a theoretical mechanical model (Section 3) of the system operating as a forced damped harmonic oscillator and the code used to calculate the power spectrum (dB) and the CWT using the seism data (Section 4) (ZIP)
