# Supplementary material for: A Robust Self-Powered Triboelectric Sensor for Risk Mitigation in Seismic Scenarios: IoT Communication and Dimensional Monitoring
Source: ACS Omega. 2026 Jun 11;11(24):35357–74. doi: 10.1021/acsomega.6c00458 (PMC13294914; doi:10.1021/acsomega.6c00458)
Supplement: Supplementary file 1 [file ao6c00458_si_001.zip › R4ListOfContents_SI2.pdf]

“The following list of contents were considered in the SI: calculation of the TENG elastic constant, different figures related to the experimental set-up, calibration curves with the TENGs, simulations of the Isc & Voc performed with Python in Google Collaborate and electrical measurements. Also, a comparison table of the D220-A4BR-1305YB piezoelectric sensor and 2D-SEISTENG transducer, seismic vs. piezoelectric sensor sensitivities and dynamic range the same as velocity and acceleration physical magnitudes. In addition, different sensor characteristics sold in the market from different companies, dominant frequencies and Power Spectral Density measured with 2D-SEISTENG of the Lorca earthquake simulated in the CEDEX (Centro de Estudios y Experimentación de Obras Públicas) vibrating table. Furthermore, a theoretical mechanical model (Section 3) of the system operating as a forced damped harmonic oscillator and the code used to calculate the power spectrum (dB) and the CWT using the seism data (Section 4).”
