# Supplementary material for: A Robust Self-Powered Triboelectric Sensor for Risk Mitigation in Seismic Scenarios: IoT Communication and Dimensional Monitoring
Source: ACS Omega. 2026 Jun 11;11(24):35357–74. doi: 10.1021/acsomega.6c00458 (PMC13294914; doi:10.1021/acsomega.6c00458)
Supplement: Supplementary file 1 [file ao6c00458_si_001.zip › Supporting Information.pdf]

SUPPORTING INFORMATION

A robust self-powered triboelectric sensor for risk mitigation in seismic scenarios: IoT communication and dimensional monitoring

José Sánchez del Río[a,b,c,*], Antonio Vázquez-López [b,d], Jorge Edison Pozo Benavides[a], Alba López Laguna[a], Martin Andolfi [a], Rafael Cascón[a], Francisco Santos Olalla[a], Sofía Paramio[a], Yolanda Ballesteros[e], Carlos Cruz[f], Vanesa Martínez[b], José Luis Jiménez[b] , José Benito Bravo Monge[g], Xiang Ao[b], and De-Yi Wang[b,*]

[a] Universidad Politécnica de Madrid (UPM), E.T.S. de Ingeniería y Diseño Industrial, C/ Ronda de Valencia 3, 28012, Madrid, Spain.

[b] IMDEA Materials Institute, C/Eric Kandel, 2, Getafe, Madrid 28906, Spain.

[c]Mechanical Engineering Department, Universidad Pontificia de Comillas, Alberto Aguilera 25, 28015, Madrid, Spain.

[d]Materials Science and Engineering Area, Escuela Superior de Ciencias Experimentales y Tecnología, University Rey Juan Carlos, Tulipán Street, 28933 Móstoles, Madrid, Spain

[e]Institute for Research in Technology, Mechanical Engineering Department, Universidad Pontificia de Comillas, Alberto Aguilera 25, 28015, Madrid, Spain.

[f]University of Alcala, Department of Electronics, Alcala de Henares, 28871, Madrid, Spain.

[g]Instituto Geográfico Nacional (IGN). Space Applications, Geophysics and Astronomy Division. General Subdirection. C/ General Ibáñez de Ibero, 3. 28003 Madrid, Spain

*Correspondence and requests for materials should be addressed to JSRS (*jose.sanchezdelrio@upm.es) and D.W. *deyi.wang@imdea.org)*

The following list of contents were considered:

1. Calculation of the TENG elastic constant. For this, spherical mass diameter, weight and TENG stiffness was needed.
2. Different figures related to the experimental set-up, calibration curves with the TENGs, simulations of the Isc & Voc performed with Python in Google Collaborate and electrical measurements were shown.
3. A comparison table of the D220-A4BR-1305YB piezoelectric sensor and 2D-SEISTENG transducer is depicted.
4. Seismic vs. piezoelectric sensor sensitivities and dynamic range was measured the same as velocity and acceleration physical magnitudes.
5. Different sensor characteristics sold in the market from different companies, dominant frequencies and Power Spectral Density measured with 2D-SEISTENG of the Lorca earthquake simulated in the CEDEX (Centro de Estudios y Experimentación de Obras Públicas) vibrating table are shown.
6. In addition, Section 3, the theoretical mechanical model of the system operating as a forced damped harmonic oscillator is described.
7. To finish, the code used to calculate the power spectrum (dB) and the CWT using the seism data is summarized in Section 4.

Section 1: Calculation of the TENG elastic constant

Calculation of the spherical mass diameter and weight:

The weight of the mass to be used for the definitive device experiments is measured using a three-digit scale. A spherical mass is chosen since no change in efficiency has been seen depending on the pressure area to cause compression of the sensor. In addition, its shape allows a fairly easy push against the rigidity of the sensor.

Table S1: Measurements made to find the weight of the spherical mass.

**Spherical mass weight**						
Meas. 1	Meas. 2	Meas. 3	Meas. 4	Meas. 5	**Average**	**Std. Dev.**
546,7 g	548,1 g	545,9 g	546,2 g	546,7 g	546,7 g	0,8 g

Next, the diameter of the spherical mass to be used is measured using a calliber:

Table S2: Measurements carried out to determine the diameter of the spherical mass.

**Spherical mass diameter**						
Meas. 1	Meas. 2	Meas. 3	Meas. 4	Meas. 5	**Average**	**Std. Dev.**
50,7 mm	50,75 mm	50,5 mm	50,75 mm	50,8 mm	50,7 mm	0,12 mm

The values to be used for the weight and diameter of the spherical mass are 550 g and 51 mm, respectively.

Calculation of the TENG stiffness:

Considering the TENG as a spring, the force that must be exerted to compress or stretch it is proportional to its displacement from the equilibrium position, Δx. The relationship between the proportionality constant k and the displacement described above is known as Hooke's law:

$$F = k\,\Delta x \quad (1)$$

To measure the constant k, the strain Δx is measured when different values of the force are applied. The sensor is subjected to a series of tests with different weights ranging from 20 grams up to 100 grams (3 tests per weight). The triboelectric sensor is placed vertically and place the weights on it, thus compressing the top sheet. All these variables together with the TENG stiffness calculated is shown in **Table S3**. As a result, the TENG has a stiffness of approximately 151 N/m.

Table S3: Summary of the measurements carried out, as well as the results obtained for the mean and standard deviation for the stiffness of the triboelectric sensor

**Mass of the weight (g)**	**Weight (N)**	**Initial length (mm)**	**Final length (mm)**	**Elongation (mm)**	**Stiffness (N/m)**
20	0.196	16	14,7	1,3	150,7
20	0.196	16	14,75	1,25	156,8
20	0.196	16	14,6	1,4	140,0
50	0.49	16	12,8	3,2	153,2
50	0.49	16	13,1	2,9	168,9

50	0.49	16	12,5	3,5	140,0
100	0.981	16	9,9	6,1	160,8
100	0.981	16	9,1	6,9	142,1
100	0.981	16	9,3	6,7	146,4
				**Mean value:**	150,9
				**Standard deviation:**	10,0

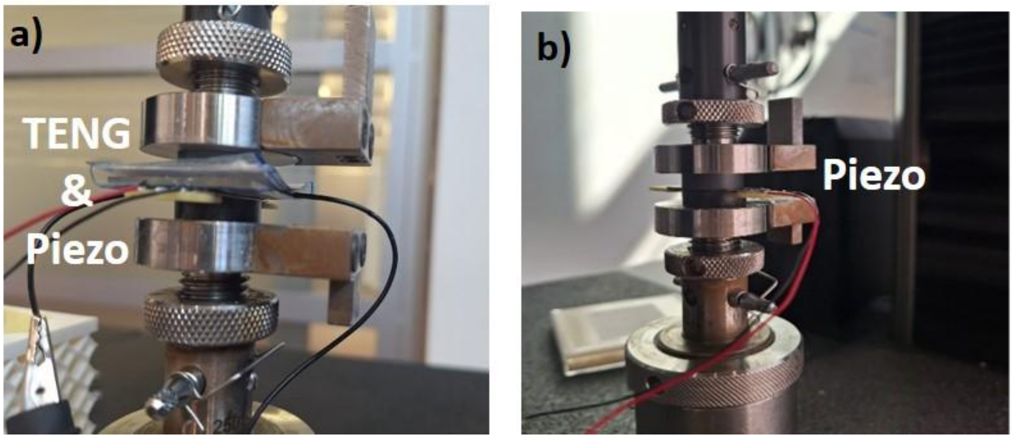

Figure S1: a) TENG and piezoelectric D220 sensors crabbed by the INSTRON jaws for TENG calibration; b) Piezoelectric sensor D220 crabbed by INSTRON jaws for calibration.

a) b)

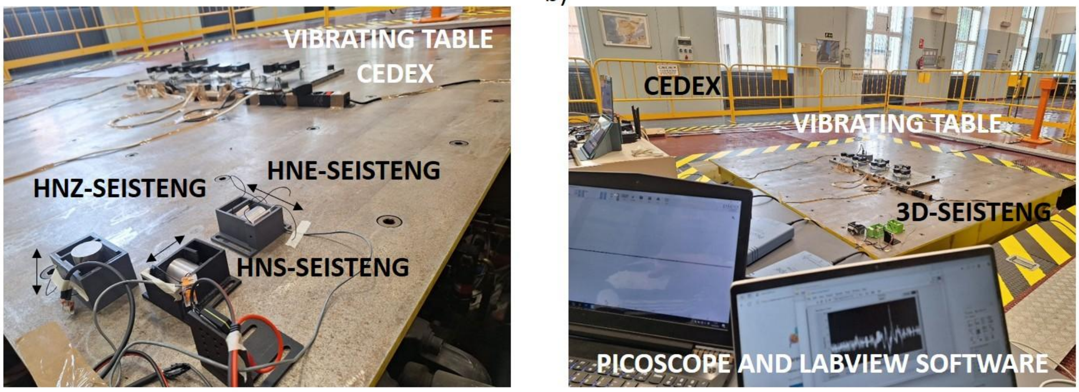

Figure S2: a) Different components of the 3D-SEISTENG fixed on the vibrating table owned by CEDEX; b) DAQ used to monitor the different simulated seism events.

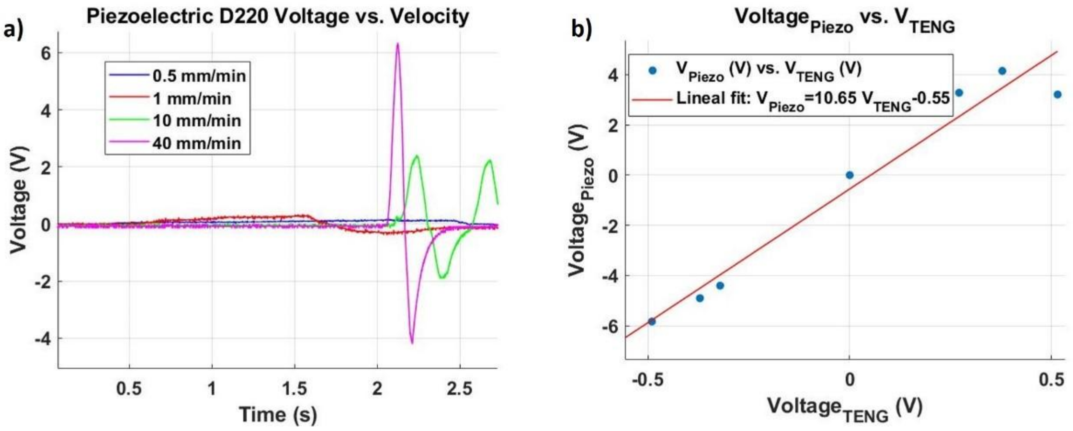

Figure S3: a) Voltage pulses generated by the piezoelectric sensor for different velocities; b) Relation between the Voltage generated by the TENG and the Voltage generated by the piezoelectric sensor for different jaw velocities.

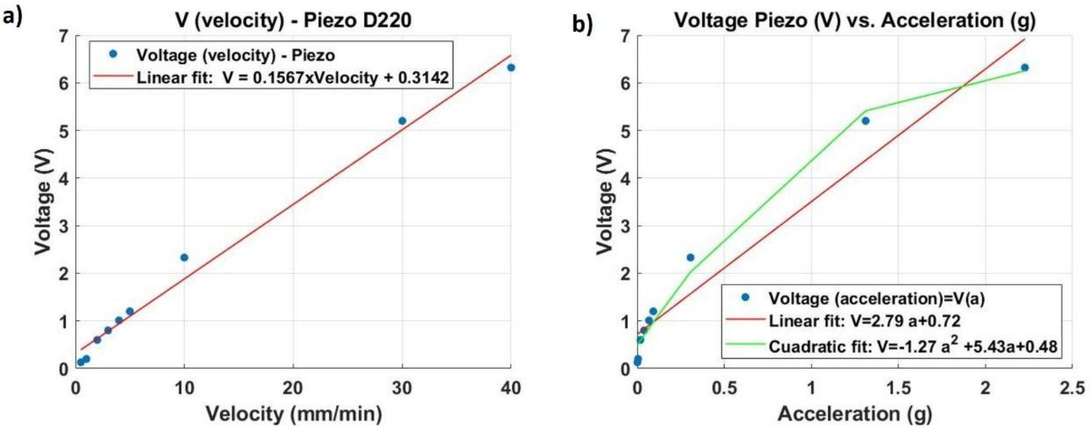

Figure S4: Calibration curves of the piezoelectric sensor D220 for different a) jaw velocities and b) acceleration velocities.

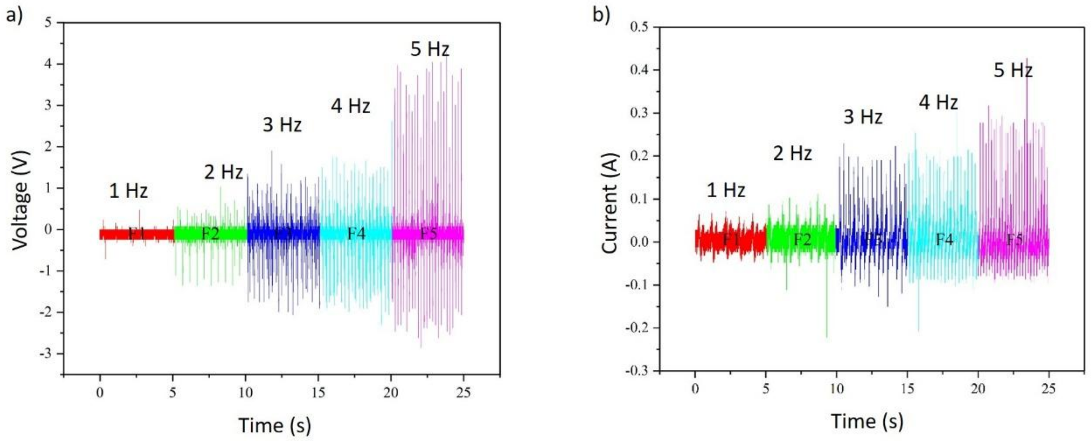

Figure S5: a) Different voltage and b) electrical current generation for the frequencies 1-5 Hz.

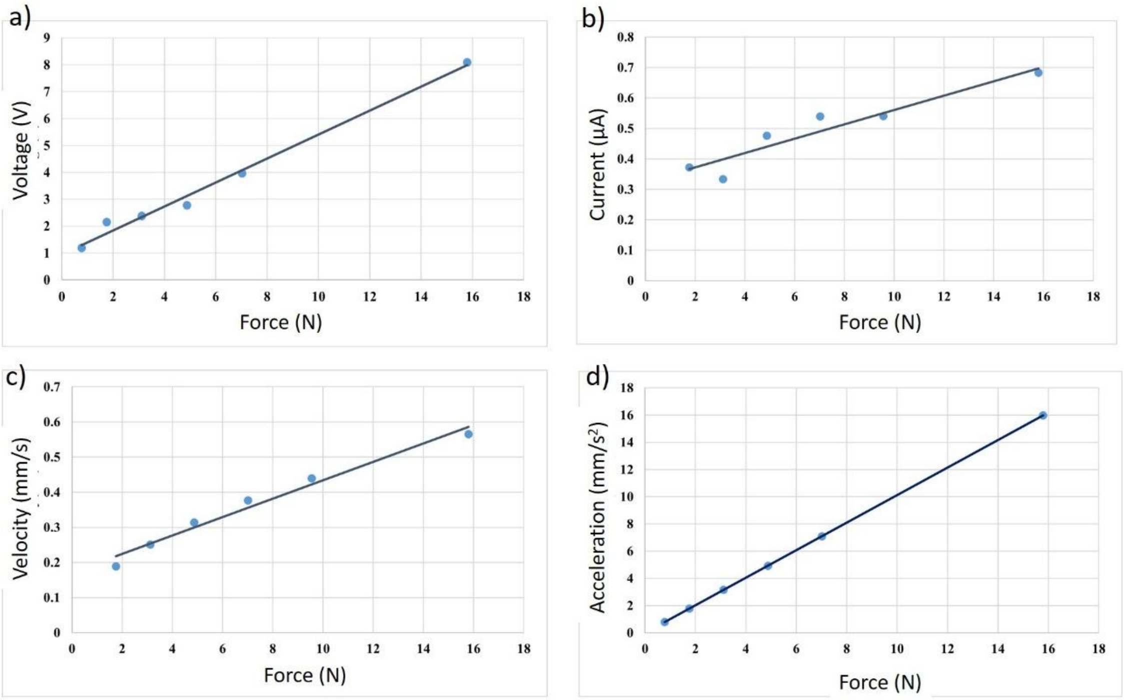

Figure S6: a) Voltage (V) vs. Force (N); b) Current (μA) vs. Force (N); c) Velocity (mm/s) vs. Force (N) and d) acceleration (mm/s) vs. Force (N).

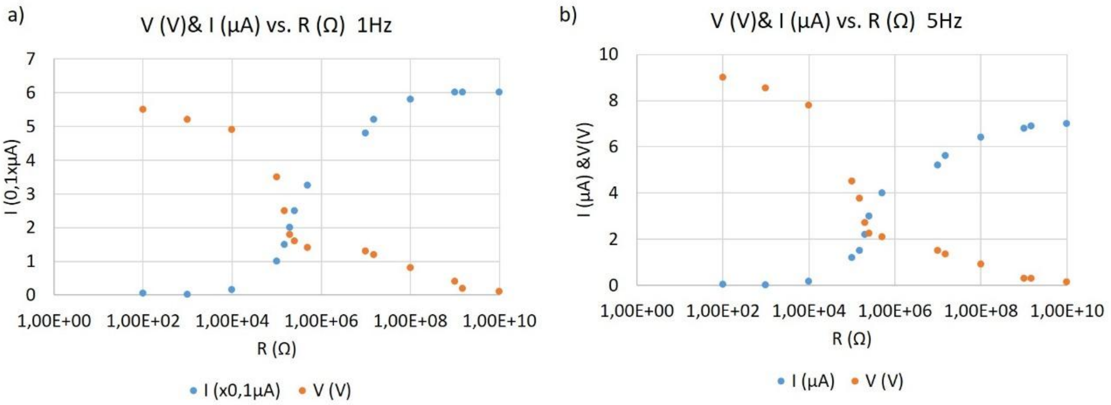

Figure S7: Voltage (V) and Current (I) curves vs. Resistance (Ω)

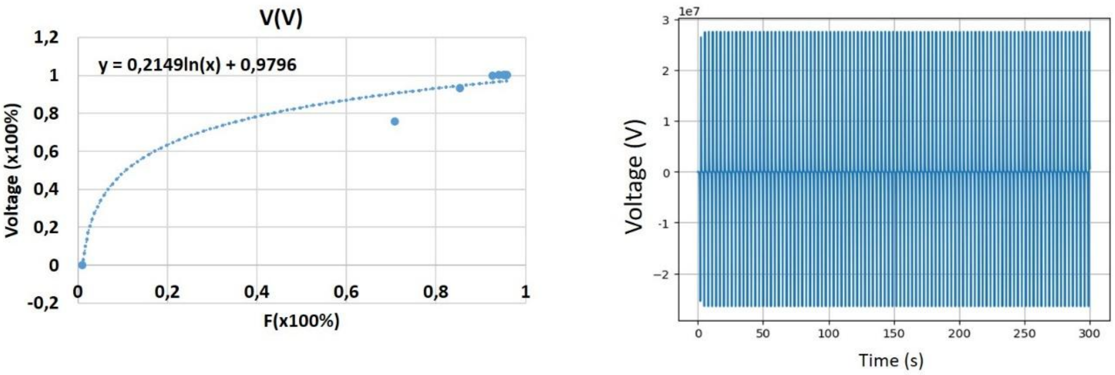

Figure S8: a) Voltage (%) vs. Force (%) simulated when TENGs are under compression and b) simulation of the voltage generated by the mechanical jaws in compression/relaxation mode for a force near to the maximum V saturated value.

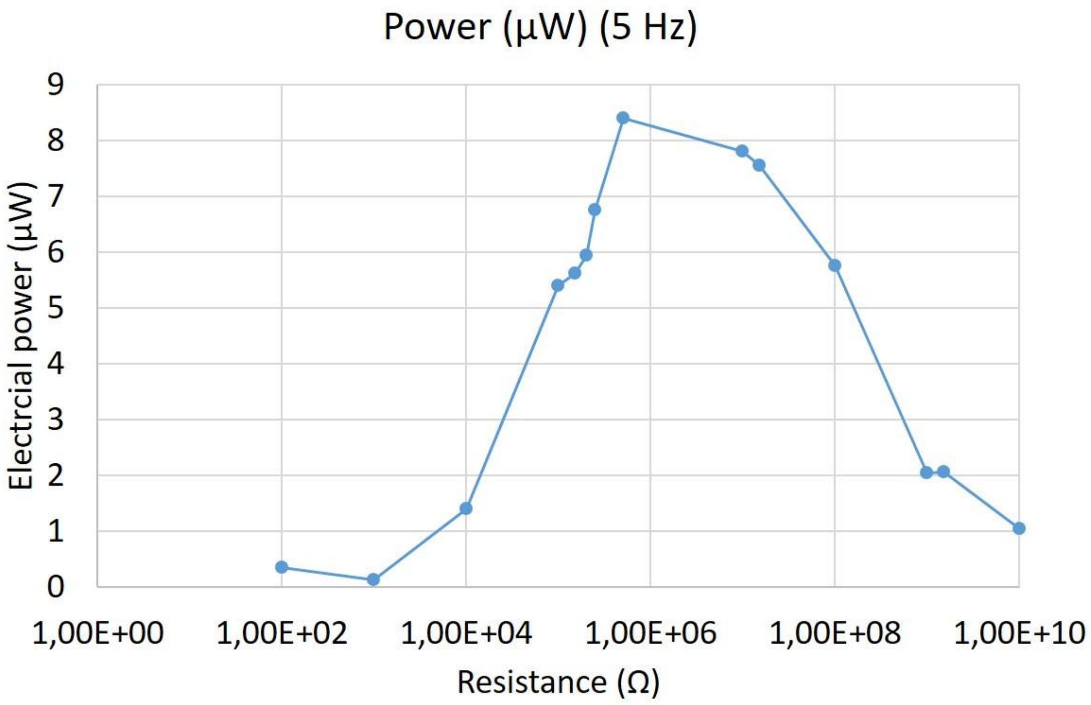

Figure S9: 2D-SEISTENG Electrical power (mW) generation for different resistance values (1 – 10 GΩ) and for the vibration table frequency of 5 Hz.

Table S4: Comparison table of the D220-A4BR-1305YB piezoelectric sensor and 2D-SEISTENG transducer.

**Characteristics**	**D220-A4BR-1305YB**	**TENG of 2D-SEISTENG**
Size (mm^3)	31.8x12.7x0.51	Under request (40x70x4 here)
Temperature range (ºC)	-60ºC – 140 ºC	20ºC-200ºC (<0ºC, no tested)
Mass (g)	12.7 g	Under request (<12,5 g if needed)
Capacitance (F)	46 nF	15 pF
Nominal Voltage out of resonance (V)	90 V	Under request (Here, Voc=911 V, R_{probe}=56 MΩ
Nominal Voltage out in resonance (V)	11.5	Usually, no V resonance
Maximum elongation (mm)	0.66	Under request (3.6 mm here)
Response time (ms)	0.64	<0.64 here
Load resistance (Ω)	2250	Under request (10^5 Ω here)
Elastic constant (k) (N/mm)	1.77	Under request (Here, 0.151 N/mm)
Sensitivity (V/m/s)/(V/g)	0.15/2.8	0.02/0.3
Sensitivity (PIEZO vs. TENG)	10.65xS_{TENG} -0.55	0.1xS_{piezo} +0.05
Sensitivity deviation (SD) (Temperature)	0.3Vm/N=3%/ºC (1 cm and 10 N)	0.014 V/ºC (1.5 %/ºC)
Sensitivity deviation (SD) (Humidity)	----	1.18
Deviation from linearity (acceleration)	1.01 V/g	10.6 V/g
Dynamic range	79.1dB; [0.01–90] V	79.2 dB; [0.1-911] V

SECTION 2: Calculation of the seismic vs. piezoelectric sensors sensitivities and dynamic range.

Regarding the Sensitivity, we can speak about sensitivity in velocity (V/m/s) and acceleration (V/g), with g=9.8 m/s^2. These sensitivities are the slopes of the curves Voltage vs. velocity (see Figure S3 a)) and Voltage vs. acceleration (Figure S3 b)).

We have included in the table the Sensitivity relation between TENG and PIEZO, according to the plot of the Figure S2 b) (Spiezo=10.65xS_{TENG} -0.55). This is necessary for the following piezo- and TENG electrical parameters.

The sensitivity deviation has been evaluated for the temperature and humidity. Regarding temperature (see Figure S10), we have calculated it by using the following formula:

$$Dev\ S_T = (Vmax_{T2} - Vmin_{T1})/(T2_{max} - T1_{max})$$

Making use of the previous equation, temperature sensitivity deviation for the TENG seismic sensor is the following: Dev S_T=(10 V-0.1 V)/(30 ºC-100 ºC)=0.14 V/ºC. Furthermore, if we divide it by the maximum (Vmax=10 V), the sensitivity deviation percentage is obtained (1.4%).

It is important to highlight that we have not evaluated TENG response for T<0ºC because we consider the seismic sensor operating at T>20ºC when they are placed in craters and (high temperatures), in countries of medium latitudes with low-high temperatures and in offices and closed places with central heating in winter. In addition, due to the difficulty of the experiment (cooling module in the DMA), we postpone measuring seismic and piezoelectric sensor sensitivities at T<20ºC for future evaluations.

Regarding the piezoelectric sensitivity deviation due to temperature, we have paid attention to the Figure 8 of the article written by C. Miclea et Al (ROMANIAN JOURNAL OF INFORMATION SCIENCE AND TECHNOLOGY Volume 10, Number 3, 2007, 243–250). Here, the temperature dependence of the voltage constants g33 and −g31 between room temperature and T=100ºC is shown. Making use of the previous equation we have the following one:

$$Dev\ S_{T,piezo} = (Vmax_{T2} - Vmin_{T1})/(T2_{max} - T1_{max})$$

$$Dev\ S_{T,piezo}(\%) = 100x(Vmax_{T2} - Vmin_{T1})/Vmax_{T2}(T2_{max} - T1_{max})$$

As Vmax=10 Vm/N at T=30 ºC, Vmin= 8 Vm/N at T=100ºC, Dev $S_{T,\,piezo}$= 100x(2/70)/10 %m/N ºC=0.3%m/N ºC. If we apply the force of 10 N and for 1 cm length, then: Dev $S_{T,\,piezo}$ (%)=3%/ºC.

Similar calculations should be performed when considering different percentages of humidity (see Figure S12).

$$Dev\ S_H = (Vmax_{H2} - Vmin_{H1})/(H2_{max} - H1_{max})\text{=(10-2.5)V/(50\%-90\%)=-0.18V/\%}$$

$$Dev\ S_H = 100x(Vmax_{H2} - Vmin_{H1})/Vmax_{H2}(H2_{max} - H1_{max})\text{=(0.18/10x100)\%/\%)=1.8}$$

Regarding the deviation of the sensitivity for the piezoelectric sensor, formula to be applied are the following:

$$Dev\ S_{H,piezo} = (Vmax_{H2,p} - Vmin_{H1,p})/(H2_{max} - H1_{max})$$

$$Dev\ S_{H,piezo}(\%) = (Vmax_{H2,p} - Vmin_{H1,p})/Vmax_{H2,p}(H2_{max} - H1_{max})$$

However, we have not found in literature any value of sensitivity for different humidity's and the set-up for its experimental evaluation in the lab is different from the TENG's and more complicated because much lower forces should be applied to the piezoelectric sensor. Next work will be focused on characterizing the humidity sensitivity deviation the same as the sensitivity at low T of the seismic sensor.

In addition, we have estimated the piezoelectric linearity deviation for acceleration (see Figure S10) by calculating the distance of furthest point of the quadratic fit from the linear one. This distance is read in the next Figure (obtained from the Figure S10)). Here below the formula is depicted and its value calculated:

$$d = \sqrt{(4,1\text{-}3,1)^2 + (1\text{-}0,8)^2} = \sqrt{1 + 0,04} = 1,01\ V/g$$

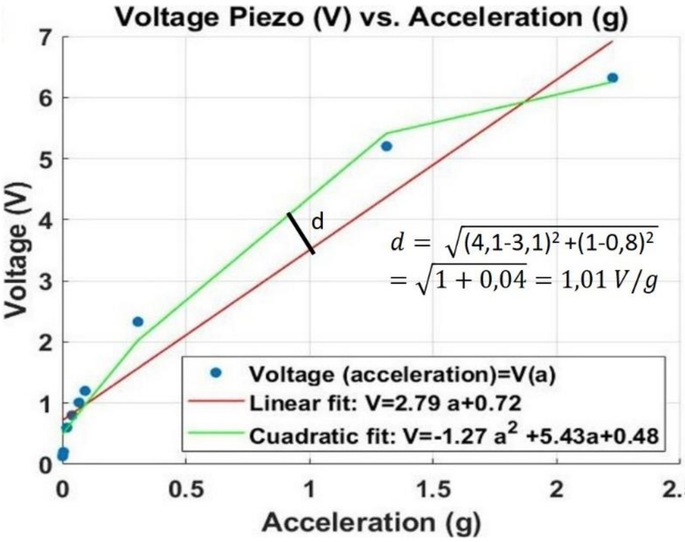

Figure S10: Calculation of the sensitivity deviation from linearity

As Spiezo=10.65xS_{TENG} -0.55, this means that the piezoelectric sensor is 10 times more sensitive than the TENG seismic sensor. As a result, d=0.1 V/g for the TENG seismic sensor.

To finish, we have calculated the dynamic range as:

$$D.R. = 20\ log_{10}\left(\frac{Vmax}{Vmin}\right)$$

For which V_{max}=Voc=911 V when measuring with a 100 MΩ probe and Vmin=0.1 V for the TENG giving a DR=79.19 dB.

If we focus on the piezoelectric sensor, V_{max}=90V, Vmin=0.01 V and DR=79.08 dB.

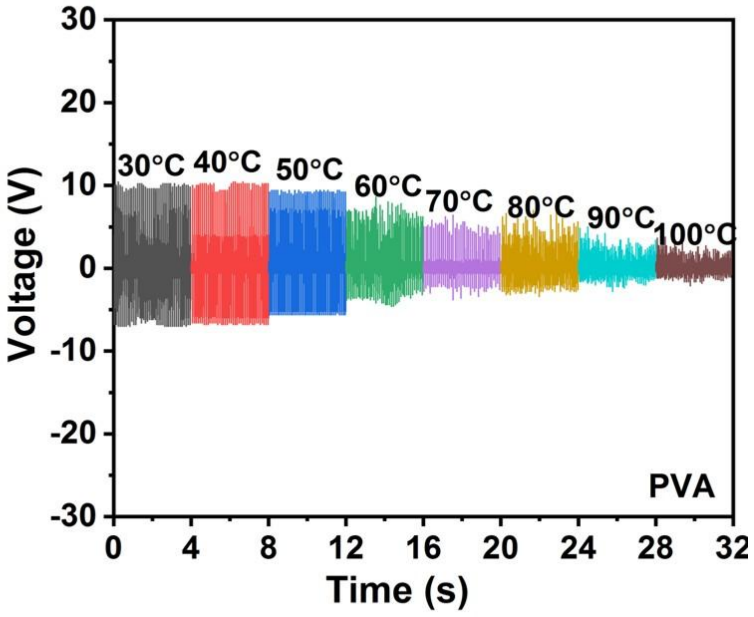

Figure S11: Voltage output vs. time for different temperatures with the PVA-based TENG.

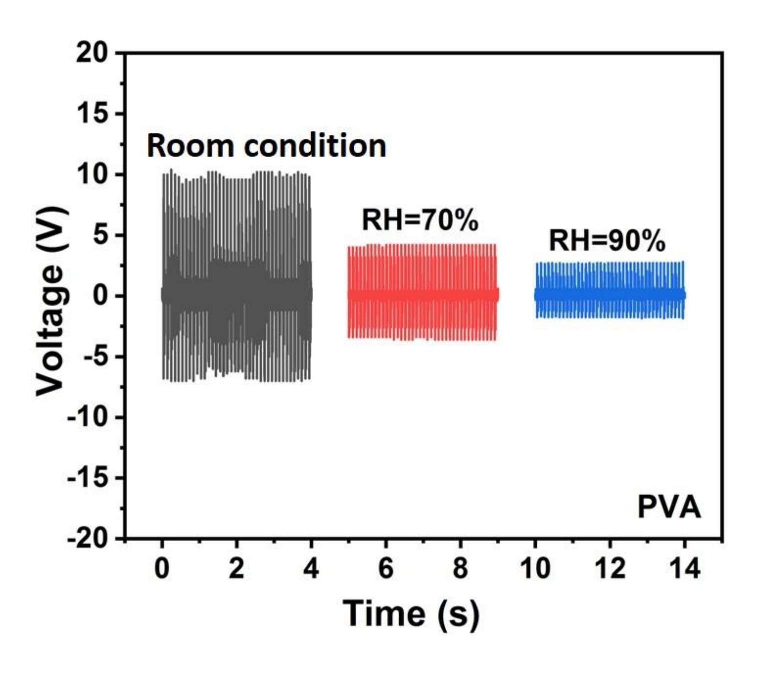

Figure S12: The comparison of humidity effect on the output voltage of PVA-based TENG for the different humidity environments.

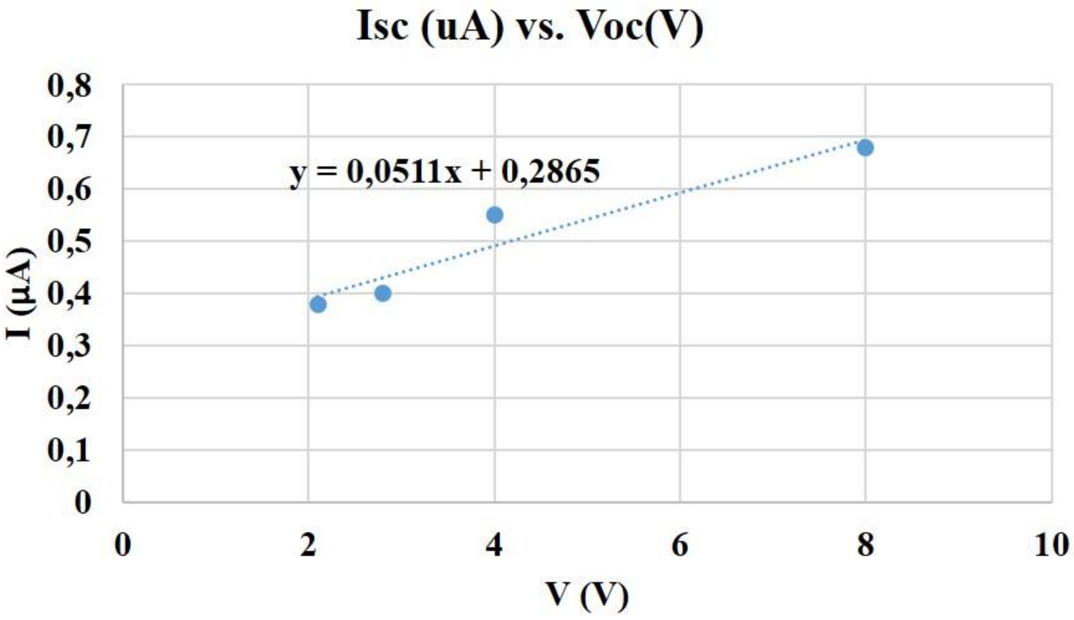

Figure S13. Relation between the Voc and Isc for the TENG seismic sensor

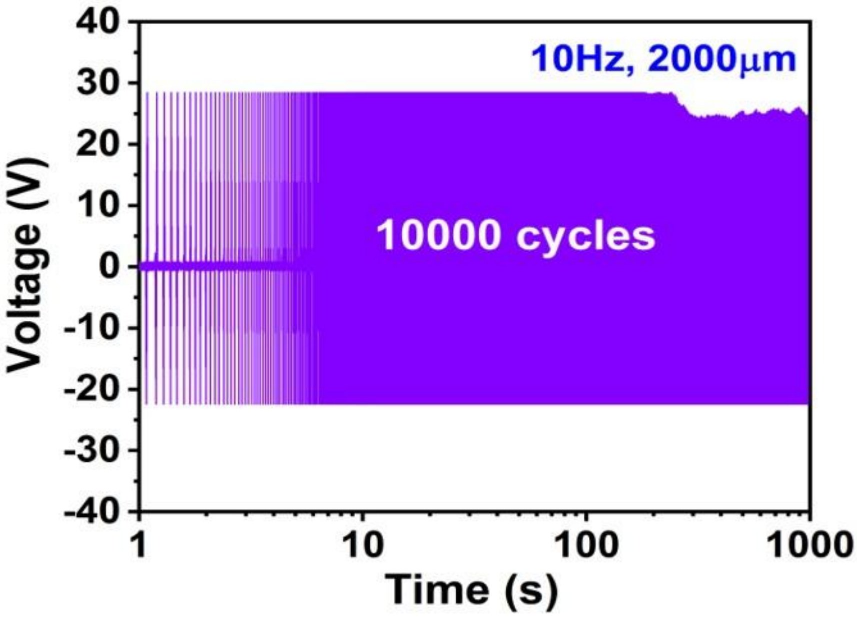

Figure S14: Stability and durability test of the best-performed TENG containing PDMS and PVA under the working condition of 10Hz and 2000 μm of separating distance over 10000 cycles.

Table S5: Characteristic different sensors sold in the market from different companies.

Company	Products	Intrinisic sensitivity	Natural/ Work freq.	Distorsion	Operating position	Oper. T	Oper. Humidity	Pressure	Portability	Noise	Power Supply
UPM	SEISTENG	20 V/m/s<br>1,6 V/g<br>100 mN<br>0,4 V<br>0,4 m/s^2	0-250 Hz	<0.1%	3 axis<br>N-S/E-W	Tmax=200ºC	100% housing<br>95% sensor	1000 bars housing	Yes	Sensor: 0%.<br>DAQ:<50 uV RMS 0-50 Hz	Sensor: NO
Sercel	Geoph.& seismometers&hydroph.	80 V/m/s<br>52 V/m/s	1,2 4.5, 5,10, 14, 15 Hz	<0.1%	Horizontal<br>Vertical	-40ºC-80ºC/200ºC	10%-90%	2,07 bars housing/1000 bars	Yes	<500 uV	Yes
Solgeo	MEMs (Accelerometers), Geoph.	2000 mV/g.<br>< 20ng/√Hz	0-400 Hz	<0.1%	3 axis<br>N-S/E-W	-40-90ºC	100% housing	0-5bars	Often left in houses	<70 uV RMS 0-50 Hz<br>Geop: 55 nm/s	Yes
MAE	Geophones	32V/m/s<br>23,6 g	4,5 Hz	<0.1%	Horizontal<br>Vertical	Room T	100% housing	1000 bars housing	Yes	<500 uV	Yes
GeoSIG	Velocity sensor/Accelerometer	±1,10,100 mm/s, 2,5-20V/g	1-315Hz/0,1 Hz-100Hz	<0,3%	X,Y,Z	-40ºC-70ºC	0-100%	1000 bars housing	Yes	<500 uV	Yes

Table S6: Dominant frequencies and Power Spectral Density measured with 2D-SEISTENG of the simulated Lorca earthquake simulated in the CXEDEX vibrating table.

**Dominant Frequencies and Power Spectral Density**	
**Frequency (Hz)**	PSD (mV²/Hz)
3.683	3.683
12.328	12.328
9.396	9.396
10.336	10.336
4.585	4.585
13.117	13.117
4.961	4.961
4.172	4.172
3.195	3.195
8.268	8.268
18.379	18.379
11.350	11.350
6.652	6.652
7.667	7.667
16.913	16.913
18.792	18.792
7.254	7.254
2.744	2.744
8.757	8.757
10.937	10.937
5.713	5.713
22.137	22.137
1.842	1.842

Section 3: Mathematical model solution

a) Model of free undamped oscillation

The oscillation does not decay over time because there are no dissipative mechanisms (viscous damping, dry friction, internal losses); therefore, the total mechanical energy of the system is conserved. Moreover, since it is treated as an unforced system, there is no external excitation term, which means that the equation has no independent terms and is thus homogeneous.

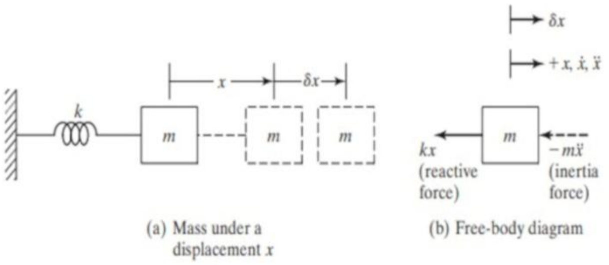

Figure S15: Representation of SEISTENG as a model of free undamped oscillation

In this type of problems, two effects will be involved:

a) Restoring effect: When the system is out of its equilibrium, an elastic force appears that tends to return it to that position. In a linear spring, the force causing the restoring effect is proportional to the displacement and opposite in direction [1]:

$$Fr = -Kx. \tag{3.1}$$

b) Inertial effect: The mass resists changes in motion, as described by Newton's second law [1]:

$$F_i = m\ddot{x}. \tag{3.2}$$

Taking x(t) as the displacement from the equilibrium position, the homogeneous differential equation of motion is:

$$m \cdot \ddot{x}(t) + k \cdot x(t) = 0. \tag{3.3}$$

Dividing by the mass m,

$$\ddot{x}(t) + \frac{k}{m}x(t) = 0. \tag{3.4}$$

Being a homogeneous differential equation with constant coefficients, a solution of the following form is proposed:

$$x(t) = e^{st}t. \tag{3.5}$$

Substituting the solution into the differential equation:

$$s^2e^{st} + \frac{k}{m}e^{st} = 0 \rightarrow e^{st}(s^2 + \frac{k}{m}) = 0. \quad (3.6)$$

Since the argument $e^{st} \neq 0$, the characteristic equation is obtained.

Solutions are the following:

$$s_{1,2} = \pm j\sqrt{\frac{k}{m}}. \quad (3.8)$$

Natural angular frequency of the system is defined as follows:

$$\omega_n = \sqrt{\frac{k}{m}}. \quad (3.9)$$

From this result, it can be inferred that, in the absence of energy dissipation mechanisms and external forces, the system oscillates indefinitely at a frequency that depends on the mass m and the stiffness k, that is, the natural frequency is the inherent frequency of the system's free motion.

b) Equation of motion with base excitation

Vibration transducers, together with other devices, are known as vibration sensors. These sensors are generally used as seismometers. A seismometer consists of a mass-spring-damper mounted on a vibrating base. The vibration results from measuring the relative displacement of the mass with respect to the table.

The instrument has a mass m, spring k, and viscous damper c, connected to a structure that moves on the ground. The mechanical quantity of interest is usually the relative displacement of the mass with respect to the base, because it drives the transducer.

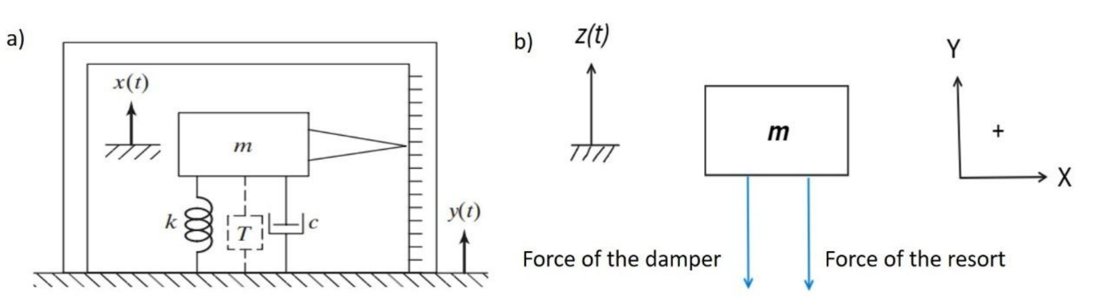

Figure S16. a) Mass and viscous damper connected to a structure inside the seismic box. b)Forces played in the system.

Where:

• y(t): displacement of the base/structure (imposed excitation),

• x(t): absolute displacement of the mass,

• z(t): relative displacement of the mass with respect to the base.

It is defined Z, as:

$$z = (x - y) \tag{3.10}$$

Base vibration is assumed as a damped harmonic motion:

$$y(t) = Y \sin \omega t, \tag{3.11}$$

And for this reason:

$$\dot{y}(t) = Y\, \omega \cos \omega t, \qquad \ddot{y}(t) = -\,Y\, \omega^2 \sin \omega t. \tag{3.12}$$

When the Second Newton law is applied, forces playing in the mass are the following:

$$\text{Damping force: } c\,(\dot{x} - \dot{y}) \tag{3.13}$$

$$\text{Resort force: } k\,(x - y) \tag{3.14}$$

Motion equation is described as:

$$m\ddot{x} + c(\dot{x} - \dot{y}) + k(x - y) = 0. \tag{3.15}$$

According to the definition of displacement z, the equation can be written as:

$$m \cdot \ddot{z} + c \cdot \dot{z} + k \cdot z = -m\,\ddot{y}. \tag{3.16}$$

If Laplace transformed is applied (3.21) and considering position initial conditions and zero velocity:

$$(m\, s^2 + c\, s + k)\, Z(s) = -m\, s^2\, Y(s). \tag{3.17}$$

If base acceleration is defined as $Abase(t) = \ddot{y}(t)$, and considering null initial conditions:

$Abase(s) = s^2\, Y(s)$,

The, a direct expression between Z(s) and Abase (s) is obtained:

$$\frac{Z(s)}{Abase(s)} = -\frac{m}{(m\, s^2 + c\, s + k)}. \tag{3.18}$$

The frequency response is obtained by evaluating the transfer function on the imaginary axis, that is, by substituting s = jω. It is defined as:

$$H(j\omega) = \frac{Z(j\omega)}{Abase(j\omega)} = -\frac{m}{(k - m\,\omega^2 + j\,c\,\omega)}. \tag{3.19}$$

To develop magnitude and phase, the numerator and denominator are separated as complex numbers:

$$N(j\omega) = 1, \quad D(j\omega) = (k - m\,\omega^2 + j\,c\,\omega). \tag{3.20}$$

c) Justification of magnitude and phase of H(jω)

For any complex number Z≠0, there exists a polar representation:

$$Z = |Z_1| e^{j\angle Z}, \tag{3.21}$$

where |Z| is its magnitude and ∠Z its argument or phase.

Si $Z_1 = |Z_1|e^{j\theta_1}$ y $Z_2 = |Z_2|e^{j\theta_2}$, entonces:

$$Z_1 Z_2 = (|Z_1||Z_2|)e^{j(\theta_1+\theta_2)}, \qquad \frac{Z_1}{Z_2} = \frac{|Z_1|}{|Z_2|}e^{j(\theta_1 - \theta_2)}. \tag{3.22}$$

From these expressions, the following relations can be obtained:

$$|Z_1 Z_2| = |Z_1||Z_2|, \qquad \left|\frac{Z_1}{Z_2}\right| = \frac{|Z_1|}{|Z_2|}e^{j(\theta_1 - \theta_2)}. \tag{3.23}$$

$$\angle(Z_1 Z_2) = \angle Z_1 + \angle Z_2, \qquad \angle(\frac{Z_1}{Z_2}) =) = \angle Z_1 + \angle Z_2. \tag{3.24}$$

In phasor analysis and in the use of complex numbers, it is common to represent the complex magnitude as (3.31). In this context, "n" is used to represent a positive real magnitude, associated with the modulus of the complex number. Since "n" > 0 and is a real number, its representation on the complex plane is located on the positive real axis without phase:

$$n = n + j0 = ne^{j0} \Rightarrow \angle(m) = 0. \tag{3.25}$$

On the other hand, placing a negative sign in front of a number is equivalent to multiplying it by −1:

$$-\mathrm{n} = (-1)\mathrm{n}. \tag{3.26}$$

Making use of the Euler identity: $e^{j\theta} = cos\theta + jsin\theta$, se tiene

$$e^{j\theta} = cos(\pi) + jsin(\pi) = -1. \tag{3.27}$$

And this gives the following relation:

$$-1 = e^{j\pi} \Rightarrow (-1) = \pi \text{ rad} = 180^{o}. \qquad (3.28)$$

Consequently, the negative sign does not alter the magnitude (the modulus remains n), but it introduces a phase shift of 180°:

$$-m = m\, e^{j\pi}. \qquad (3.29)$$

Meaning that,

$$H(j\omega) \;=\; \left|-m \cdot \frac{N(j\omega)}{D(j\omega)}\right| = m\left|\frac{N(j\omega)}{D(j\omega)}\right|, \qquad \angle H \;=\; \pi \;+\; \angle N \;-\; \angle D. \qquad (3.30)$$

Magnitude

The magnitude is the result of the division between the magnitudes of the numerator and denominator:

$$\mathrm{N}(\mathrm{j}\omega) \;= 1, \quad \mathrm{D}(\mathrm{j}\omega) \;= (\mathrm{k} \;-\; \mathrm{m}\,\omega^2 \;+\; \mathrm{j}\,\mathrm{c}\,\omega). \qquad (3.31)$$

$$\mathrm{N}(\mathrm{j}\omega)| \;=\; 1, \quad |\mathrm{D}(\mathrm{j}\omega)| \;=\; \sqrt{(\mathrm{k} \;-\; \mathrm{m}\,\omega^2)^2 \;+\; (\mathrm{c}\,\omega)^2}, \qquad (3.32)$$

$$|\mathrm{H}(\mathrm{j}\omega)| \;=\; \mathrm{m}\,\frac{1}{\sqrt{(\mathrm{k} \;-\; \mathrm{m}\,\omega^2)^2 \;+\; (\mathrm{c}\,\omega)^2}}. \qquad (3.33)$$

Its equivalent in frequency expressed in cycles is:

$$|\mathrm{H}(\mathrm{j}2\mathrm{f}\pi)| \;=\; \mathrm{m}\,\frac{1}{\sqrt{(\mathrm{k} \;-\; \mathrm{m}\,(2\mathrm{f}\pi)^2)^2 \;+\; (\mathrm{c}\;2\mathrm{f}\pi)^2}}. \qquad (3.34)$$

Phase

The phase of a quotient is the difference of phases, and the “–” sign provides a 180° shift:

$$\angle\mathrm{H}(\mathrm{j}\omega) \;=\; 180° \;+\; \angle(1 + 0\mathrm{j}) \;-\; \angle((\mathrm{k} \;-\; \mathrm{m}\,\omega^2) \;+\; \mathrm{j}\,\mathrm{c}\,\omega). \qquad (3.35)$$

For a complex number a + jb (with a ≠ 0), a common way to express its angle:

$$\angle(a + j\,b) = arctan(\frac{b}{a}). \tag{3.36}$$

Its equivalent in frequency expressed in cycles is:

$$\angle(1) = arctan(0) = 0°. \tag{3.37}$$

$$\angle\big((k - m\,\omega^2) + j\,c\,\omega\big) = arctan\left(\frac{c\,\omega}{k - m\omega^2}\right). \tag{3.38}$$

$$\angle \mathrm{H}(\mathrm{j}\omega) = 180° - \arctan\left(\frac{\mathrm{c}\,\omega}{\mathrm{k} - \mathrm{m}\,\omega^2}\right). \tag{3.39}$$

Its equivalent in frequency expressed in cycles is:

$$\angle \mathrm{H}(\mathrm{j}2\mathrm{f}\pi) = 180° - \arctan\left(\frac{\mathrm{c}\,2\mathrm{f}\pi}{\mathrm{k} - \mathrm{m}\,(2\mathrm{f}\pi)^2}\right). \tag{3.40}$$

d) Implementing the model:

The equivalent dynamic model of the prototype is similar to that presented in the equation of motion with base excitation:

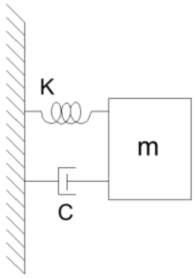

Figure S17: Schematics of SISTENG mechanical model

Relative displacement is defined as z(t) = x(t) - y(t), where x(t) is the absolute displacement of the mass and y(t) is the displacement of the base. In the frequency domain, the response function is used:

$$\mathrm{H}(\mathrm{j}\omega) = \frac{Z(\mathrm{j}\omega)}{Abase(\mathrm{j}\omega)}$$

For the base excitation, the frequency response function can be expressed as:

$$\frac{Z(s)}{Abase(s)} = -\frac{m}{(m\,s^2 + c\,s + k)}. \tag{3.41}$$

The magnitude and phase are defined as:

$$|\mathrm{H}(\mathrm{j}2\mathrm{f}\pi)| = \mathrm{m}\,\frac{1}{\sqrt{(\mathrm{k} - \mathrm{m}\,(2\mathrm{f}\pi)^2)^2 + (\mathrm{c}\,2\mathrm{f}\pi)^2}}. \tag{3.42}$$

$$\angle\mathrm{H}(\mathrm{j}2\mathrm{f}\pi) = 180° - \arctan\left(\frac{\mathrm{c}\,2\mathrm{f}\pi}{\mathrm{k} - \mathrm{m}\,(2\mathrm{f}\pi)^2}\right). \tag{3.43}$$

It is worked with a fraction of the critical damping ζ=0.005, typical of steel springs.

Applying the magnitude and phase equations, with the conditions of K=150.9 N/m and a mass m=0.5467 kg. The viscous damping coefficient is obtained using the following expression:

$$\mathrm{c} = 2\ \zeta\sqrt{2 \cdot k\,m} \tag{3.44}$$

$$\mathrm{c} = 2\ 0.005\sqrt{150{,}9 \cdot 0{,}5} = 0{,}00868\ \mathrm{N} \cdot \mathrm{s/m}$$

By substituting values into the magnitude and phase equations, the following formulations are obtained:

$$|\mathrm{H}(\mathrm{j}2\mathrm{f}\pi)| = 0.5\,\frac{1}{\sqrt{(150{,}9 - 0{,}5467\,(2\mathrm{f}\pi)^2)^2 + (2 \cdot 0{,}00868\,\mathrm{f}\pi)^2}}. \tag{3.45}$$

$$\angle H(j2\mathrm{f}\pi) = 180° - arctan\left(\frac{2 \cdot 0{,}00868\mathrm{f}\pi}{150{,}9 - 0{,}5467\,(2\mathrm{f}\pi)^2}\right). \tag{3.46}$$

When graphing equations (3.41 and 3.42) in Matlab, we get:

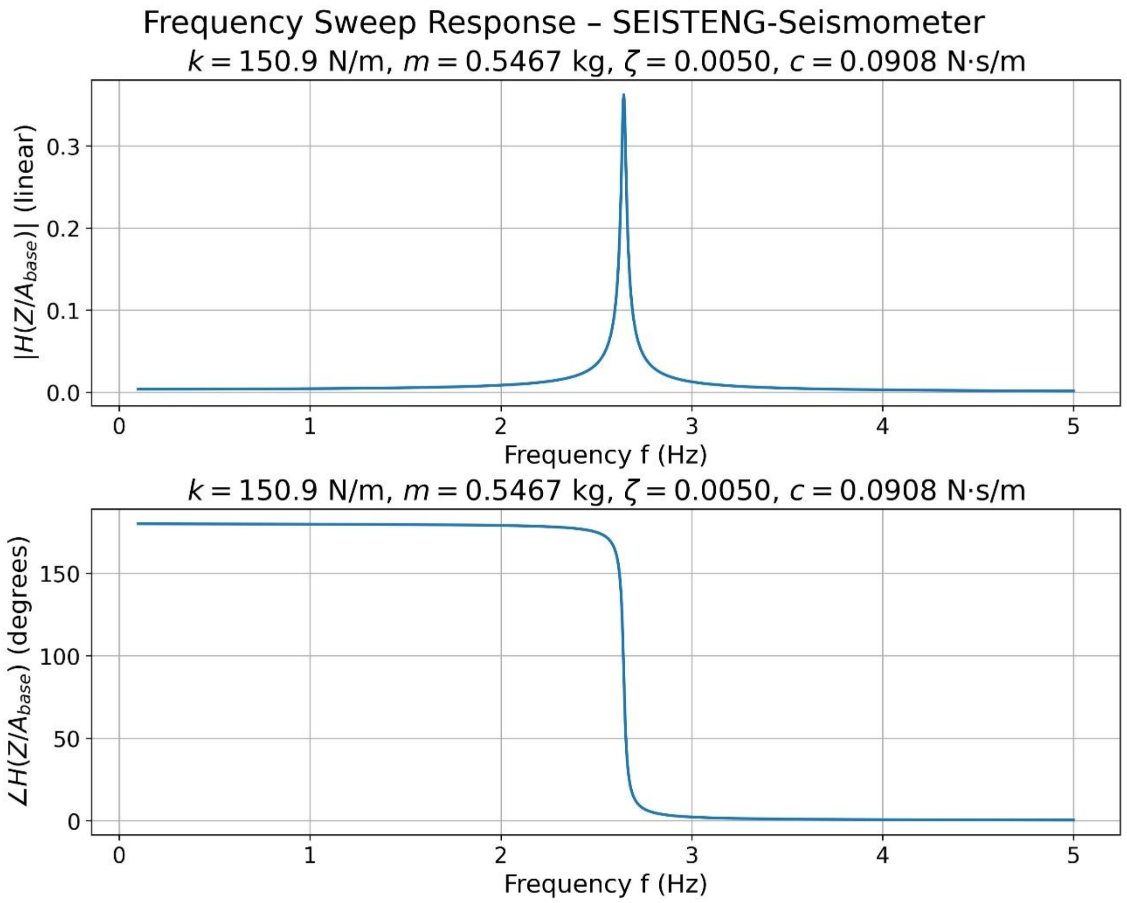

Figure S18: Solutions of the SEISTENG mechanical damping model.

e) Analysis with the graphs

The relative displacement maintains an almost constant phase lag with respect to the angular acceleration of the platform, at an angle close to 180º, up to approximately 2 Hz. For higher frequencies, a significant change in the phase lag begins to be observed, indicating that the system stops behaving as an almost static relationship and enters a range where dynamic effects are dominant. The system's resonance is at 2.64 Hz.

On the other hand, the magnitude starts to increase with frequency due to approaching the system's resonance, causing the mass-spring-damper assembly to act as a mechanical amplifier, increasing the amplitude of the relative displacement in response to the base excitations. In the frequency range from 1 Hz to 2.64 Hz, this increase becomes significant; for this reason, the measurements taken with the TENG show peaks of higher amplitude than those recorded by the used accelerometer as reference.

Although the behavior of the system can be modeled in segments (free motion and contact), in the regime of external excitation with high frequencies and low amplitudes, the system can be approximated by a single degree of freedom (SDOF) model, as long as the clearance between the

mass and the TENG elastomer is very small. Under these conditions, contact with the elastomer tends to be practically continuous, so that switching between phases becomes negligible and the system can be linearized around the operating point, resulting in an equivalent mass–spring–damper model, where the effective stiffness is associated with the elastic deformation of the elastomer.

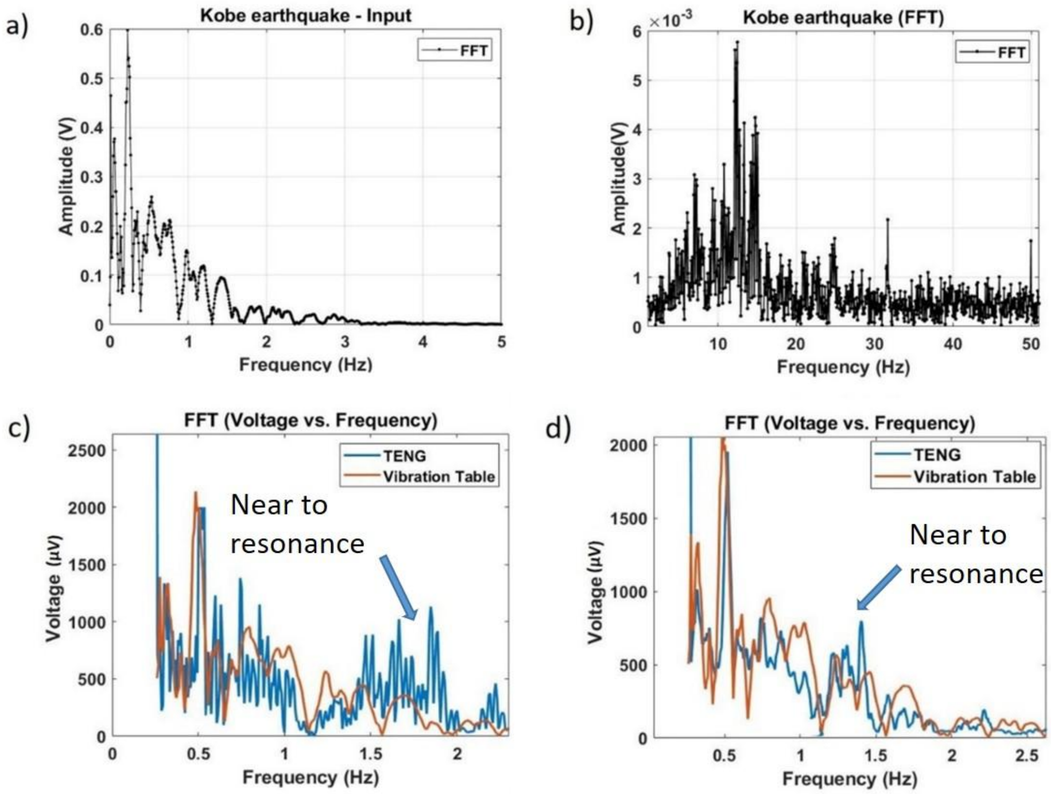

Figure S19: a) Excitation frequency (Kobe). B) 2D-SEISTENG raw data. C) Frequency filtering, see higher amplitude of SEISTENG near to resonance. D) SEISTENG signal after smoothing.

SECTION IV: Code used to calculate the power spectrum (dB) and the CWT using the seism data. Explanation.

```
%Combine signals


%basnpass - no sale bien del todo. Ver frecuencias
readsac
TENG=ans
readsac
vibratingTable=ans


Ts=readmatrix("seismicstation_ts.csv")
csvwrite("vibratingTable.csv",vibratingTable)

vibratingTableTs = Ts(1)
vibratingTable =
readtimetable("vibratingTable.csv","SampleRate",1/vibratingTableTs)
plot(vibratingTable.Time,vibratingTable.Var1)


%SIGNALS COMPARISON
TENGTs = Ts(2)
csvwrite("TENG.csv",TENG)
TENG = readtimetable("TENG.csv","SampleRate",1/TENGTs)
plot(TENG.Time,TENG.Var1)

%Task 2
vibratingTable=normalize(vibratingTable)
TENG=normalize(TENG)

plot(vibratingTable.Time,vibratingTable.Var1)
hold on
plot(TENG.Time,TENG.Var1)
hold off
legend("VIBRATINGTABLE","TENG")
xlim(seconds([2800 3500]))

%Task 1
quakes=synchronize(vibratingTable,TENG)


quakes.Properties.VariableNames = ["VIBRATINGTABLE" "TENG"]


[p,f] = pspectrum(quakes);
semilogx(f,db(p,"power"))
legend("VIBRATINGTABLE","TENG")
xlabel("Frequency (Hz)")
ylabel("Power Spectrum (dB)")
```

```
%Task 1
xline(0.1)


[p,f] = pspectrum(quakes);
figure
semilogx(f,db(p,"power"))
legend("VIBRATINGTABLE","TENG")
xlabel("Frequency (Hz)")
ylabel("Power Spectrum (dB)")
```

Description:

1. Calculation of the Power Spectrum (FFT-based) and CWT

The power spectrum shown in Figures 9 and 10 was obtained using standard Fast Fourier Transform (FFT) procedures implemented in MATLAB's Signal Processing Toolbox. The following steps have now been explicitly described in the manuscript:

- Acquisition of the raw voltage time-series from both the accelerometer and the TENG.
- Application of a low band-pass filter (cut-off 10 mHz) and a moving-mean smoothing function to reduce high-frequency noise not associated with the seismic excitation.
- FFT computation using MATLAB's FFTfunction.
- Amplitude normalization based on the length of the signal.
- Conversion of the resulting amplitude spectrum to power spectral density in decibels (dB) using 20·log10.
- Construction of the frequency axis according to the sampling frequency used during acquisition.

These steps ensure that the FFT-derived spectra for both sensors are directly comparable.

2. Calculation of the CWT (Scalogram)
   The CWT scalograms presented in Figures 9 and 10 were computed using MATLAB's cwt function with a Morlet (analytic Morse) wavelet in the Signal analyzer tool, which provides good time–frequency localization for low-frequency seismic signals. The workflow included:

- Using the filtered time-domain signal as input.
- Running MATLAB's CWT function with the sampling frequency.
- Extracting the absolute magnitude of the wavelet coefficients.
- Plotting the resulting time–frequency–amplitude representation, where the amplitude corresponds to the magnitude of the wavelet transform.

This method is commonly used in seismic analysis to show the temporal evolution of dominant frequency components, and it is consistent with the spectra shown in the manuscript.

3. Clarifications:

To fully address the reviewer's concern, we have added:

- a subsection in the Methods describing the FFT and CWT procedures
- the filtering parameters applied before spectral analysis, and
- the wavelet type, sampling frequency, and MATLAB functions used.

These additions ensure full reproducibility of the spectral and time–frequency analyses shown in Figures 9 and 10.
